# Epidemiology and impact of methicillin-sensitive *Staphylococcus aureus* with β-lactam antibiotic inoculum effects in adults with cystic fibrosis

**DOI:** 10.1128/aac.00136-23

**Published:** 2023-11-15

**Authors:** J. Svishchuk, K. Ebbert, B. Waddell, C. Izydorczyk, N. Acosta, R. Somayaji, H. R. Rabin, C. L. Bjornson, L. Lisboa, D. B. Gregson, J. M. Conly, M. G. Surette, M. D. Parkins

**Affiliations:** 1 Department of Microbiology, Immunology, and Infectious Diseases, University of Calgary, Calgary, Alberta, Canada; 2 Department of Pediatrics, University of Calgary, Calgary, Alberta, Canada; 3 Department of Medicine, University of Calgary and Alberta Health Services, Calgary, Alberta, Canada; 4 Department of Pathology and Laboratory Medicine, University of Calgary and Alberta Health Services, Calgary, Alberta, Canada; 5 Department of Biochemistry and Biomedical Sciences, McMaster University, Hamilton, Ontario, Canada; The Peter Doherty Institute for Infection and Immunity, Melbourne, Victoria, Australia

**Keywords:** MSSA, cefazolin, piperacillin-tazobactam, exacerbation, antimicrobial resistance, bacteremia, bronchiectasis, *blaZ*

## Abstract

*Staphylococcus aureus* is the most prevalent cystic fibrosis (CF) pathogen. Several phenotypes are associated with worsened CF clinical outcomes including methicillin-resistance and small-colony-variants. The inoculum effect (IE) is characterized by reduced β-lactam susceptibility when assessed at high inoculum. The IE associates with worse outcomes in bacteremia and other high-density infections, and may therefore be relevant to CF. The prevalence of IE amongst a CF cohort (age ≥18 years), followed from 2013 to 2016, was investigated. Yearly methicillin-sensitive *S. aureus* (MSSA) isolates were screened at standard (5 × 10^5^ CFU/mL) and high (5 × 10^7^ CFU/mL) inoculum against narrow-spectrum anti-Staphylococcal β-lactams and those with anti-pseudomonal activity common to CF. A ≥ 4-fold increase in minimum inhibitory concentration between standard and high inoculum defined IE. Isolates underwent *blaZ* sequencing and genotyping and were compared against published genomes. Fifty-six percent (99/177) of individuals had MSSA infection. MSSA was observed at ≥10^5^ CFU/mL in 44.8% of entry sputum samples. The prevalence of the IE was 25.0%-cefazolin; 13.5%-cloxacillin; 0%-meropenem; 1.0%-cefepime; 5.2%-ceftazidime; and 34.4%-piperacillin-tazobactam amongst baseline MSSA isolates assessed. *blaZ* A associated with cefazolin IE (*P* = 0.0011), whereas *blaZ* C associated with piperacillin-tazobactam IE (*P* < 0.0001). Baseline demographics did not reveal specific risk factors for IE-associated infections, nor were long-term outcomes different. Herein, we observed the IE in CF-derived MSSA disproportionally for cefazolin and piperacillin-tazobactam and this phenotype strongly associated with underlying *blaZ* genotype. The confirmation of CF being a high density infection, and the identification of high prevalence of MSSA with IE in CF supports the need for prospective pulmonary exacerbation treatment studies to understand the impact of this phenotype.

## INTRODUCTION

*Staphylococcus aureus* (SA) is the most prevalent cystic fibrosis (CF) pathogen isolated from >50% of persons with CF (pwCF) (1, 2). This has increased by ~20% in the last 30 years (1, 3). Importantly, CF outcomes are known to be worsened amongst individuals infected with *S. aureus* isolates with specific phenotypes including methicillin-resistant *S. aureus* (MRSA) (4) and small-colony-variants (SCVs) (5).

An increasingly relevant phenotype of methicillin-sensitive *S. aureus* (MSSA) is the potential to have reduced susceptibility to β-lactam antibiotics when present at high concentrations/inoculums (6). This “*inoculum effect”* (IE) is predominately attributable to the production and accumulation of a β-lactamase, BlaZ. BlaZ is well-known to hydrolyze penicillin, but can also exert weak hydrolytic activity on a broad range of β-lactams when abundantly present, reducing efficacy (6–20). Owing to clinical equivalence with isoxazole penicillins and reduced toxicity, cefazolin has become the first-line therapy for invasive-MSSA (21, 22). Accordingly, the IE has been studied in the greatest depth with cefazolin. In particular, the cefazolin IE has been well studied in the context of bacteremia, where its prevalence ranges from 13% to 58%(10–12, 15, 16). The cefazolin IE phenotype has been associated with 2.65-fold increased 30-day mortality in bacteremic cefazolin-treated individuals (16). Treatment failure has likewise been described in other high-inoculum infections (i.e., endocarditis and osteomyelitis) (6, 10–12, 23, 24).

The prevalence and impact of MSSA-exhibiting IEs have not been studied in CF. This is of relevance given that CF poses the risk of high-density infections. In expectorated sputum, MSSA may be present at densities as high as ≥10^6^- 10^8^ CFU/mL (25, 26). Owing to the importance of MSSA in pwCF, we sought to: (1) determine the prevalence and molecular epidemiology of IE-positive MSSA (against a range of CF-relevant β-lactams), (2) establish the molecular basis and whether the IE is intrinsic to individual strains, and (3) determine if patient characteristics or long-term clinical outcomes vary with IE.

## MATERIALS AND METHODS

### Patients and MSSA strains

The Calgary Adult CF Clinic (CACFC) is a regional program encompassing all of Southern Alberta, Canada, caring for pwCF (*n* ~ 220) ≥18 years and older. This is the fourth-largest adult CF clinic in Canada. All sputum-derived pathogens from pwCF attending the CACFC are maintained in a prospectively inventoried biobank (27). We included all pwCF attending the CACFC with clinically confirmed CF diagnoses and ≥1 MSSA-positive culture between 1 January 2013 and 31 December 2016. The first available MSSA per patient at study entry, and then yearly from 2013 to 2016, when available, were included. Individuals with prior lung transplantation were excluded if this occured prior to the study period or censured at the time thereof.

### Susceptibility testing

The first MSSA per pwCF between 2013 and 2016 was tested for susceptibility with anti-Staphylococcal β-lactams; cefazolin and cloxacillin, and those administered in the management of CF pulmonary exacerbations (PEx; where Gram-negative co-infections abound); cefepime, ceftazidime, meropenem, and piperacillin-tazobactam. Subsequent yearly isolates were tested only with cefazolin, meropenem, and piperacillin-tazobactam, following observed trends. Minimum inhibitory concentrations (MICs) were reported for each isolate at standard inoculum (SI; 10^5^ CFU/mL) and high inoculum (HI; 10^7^ CFU/mL) and were used to define IEs (Supplement).

### Definitions for the IE

We assessed two common definitions described in the literature for IE:

#### Definition 1/“The inoculum effect” (IE)

An isolate with ≥4 fold difference in MIC at SI (10^5^ CFU/mL) versus HI (10^7^ CFU/mL) (9, 15).

#### Definition 2/“The pronounced inoculum effect” (pIE)

An isolate susceptible at SI but non-susceptible at HI, using 2009 Clinical and Laboratory Standard Institute (CLSI) breakpoints (28, 29).

### Molecular analysis

The first isolate from each pwCF underwent *blaZ* identification via Sanger sequencing. All MSSA isolates were strain-typed by pulsed-field gel electrophoresis, and multilocus sequence type (MLST) was inferred (Supplement). The comprehensive antibiotic resistance database (CARD, https://card.mcmaster.ca/analyze/rgi) (30), and BLAST were used to subtype *blaZ* into types A, B, C, and D (9, 12, 15, 19, 31) (Supplement).

To understand if *blaZ* allele types in our cohort were broadly representative, we compared our data with that obtained from other cohorts. We performed analysis of *blaZ* types from published non-CF cohorts where *blaZ* alleles were reported, and from published studies including CF-derived MSSA using available *de novo* assemblies via custom Python scripts (Supplement).

### Clinical data and outcomes

Clinical characteristics and demographics were collected retrospectively by chart review. Yearly PEx requiring parenteral antibiotics (as per Fuchs criteria (32)) were documented. The number of sputum cultures collected, those yielding MSSA, MRSA, and SCV *S. aureus*, and all co-infections were recorded. Persistent MSSA was defined as an isolate being recovered during a year in which ≥ 50% of sputum cultures were MSSA+. Participant status was defined on 31 December 2016 (or at study exit) as “ongoing follow-up,” “moved/transferred,” “lung transplantation,” or “death.” The primary clinical outcome of interest was the rate of lung function decline (FEV_1_ decline (33)), with secondary outcomes including PEx frequency, change in BMI, and risk of death/lung transplantation.

### Statistical analysis

Fisher’s exact probability tests were performed for comparisons of categorical variables (1-sided). The normality of distribution was assessed using the Shapiro-Wilk test. Non-normally distributed variables were reported as medians with interquartile ranges (IQR) and compared via the Mann-Whitney rank-sum test. Normally distributed variables were reported as means with standard deviation (SD) and compared using the *t*-test. For each comparison, *p*-values were reported with odds ratios (OR) or risk ratios (RR) and 95% confidence intervals (CI), when significant. An alpha of 0.05 was used for significance in all data sets including <20 comparisons. Alpha was Bonferroni-corrected (0.05/number of comparisons) and noted when relevant for data sets with ≥20 comparisons. Assessing the similarity of our data to published cohorts was performed using two-proportion Z-score tests, while MSSA and *Pseudomonas aeruginosa* density comparisons were performed via the Spearman rank correlation test. Rates of FEV_1_ and BMI decline were calculated by dividing the sum of all values by each patient’s time in the study. Patients with only one FEV_1_ or BMI value were excluded from decline analyses. Statistical tests were performed using STATA version 17.0 (College Stn., TX).

## RESULTS

### Patient population and MSSA bacteriology

Between 2013 and 2016, 177 pwCF met study criteria and submitted 2,045 sputum samples. MSSA was cultured in 38.3% (793/2,045) of samples, in 99 (55.9%) pwCF. Participants had a mean 15.1 submitted cultures (SD: 13.2), with a mean of 4.3/year (SD: 0.46). MSSA was recovered from the CACFC biobank in 96/99 of these individuals. The three patients not included did not differ with respect to age (*P* = 0.9828) or FEV_1_ (*P* = 0.7630) and had only one isolate each. Amongst the 96 participants, 16 left the study prematurely; 14 transferred to another clinic, one received lung transplantation, and one died. The median age at entry was 25.4 years (IQR: 21.3–34.8) and 51.0% (49/96) were male. Eighty-four percent (81/96) had ≥1 F508del mutation. Median predicted FEV_1_ was 71.0% (IQR: 51.5–86.0) and FVC, 91.5% (IQR: 75.5–103) (Table E4).

In their first year, 62.5% of pwCF (60/96) had persistent MSSA infection (Table E5). Not all individuals had MSSA-positive samples each year—the median yearly isolates/participant was 2 (IQR: 1–3), for a total of 223 isolates. All 96 participants had a first yearly isolate (2013–2016), 70-a second (2014–2016), 40-a third (2015–2016), and 17-a fourth (2016) included. Median MSSA density was 10^5^ CFU/mL (IQR: 10^4^–10^6^CFU/mL) amongst first isolates. Importantly, MSSA exceeded ≥10^5^ CFU/mL in 44.8% (43/96) of initial samples. Those with persistent MSSA infection had higher mean sputum density than those without (10^5.4^ CFU/mL, SD: 10^1.12^, versus 10^4.4^ CFU/mL, SD: 10^1.18^, *P* = 0.0001). Of patients infected with MSSA, 15.6% were co-infected with MRSA, 1.0% with SCV+ MSSA, and 67.7% with *P. aeruginosa*.

### MSSA susceptibility testing

Susceptibility testing at SI and HI for cefazolin, cefepime, meropenem, ceftazidime, piperacillin-tazobactam, and cloxacillin are outlined in Table 1 for first isolates (*n* = 96). Piperacillin-tazobactam showed the highest prevalence of IEs, followed by cefazolin. As expected, the IE was infrequent with cloxacillin, cefepime and ceftazidime, and absent with meropenem. The pIE was less common than the IE and was predominantly observed with piperacillin-tazobactam. The occurrence of IE or pIE with one antibiotic did not associate with an increased likelihood of IE/pIE with other β-lactam antibiotics (data not shown). Individuals with persistent MSSA were not more likely to have an IE to any antibiotics tested, nor was higher MSSA density associated with any IE (data not shown). As IEs were more common than pIEs, these were the primary focus of further analyses.

**TABLE 1:** Prevalence of the inoculum effect (IE) and pronounced inoculum effect (pIE), and MIC_50_ and MIC_90_ from 96 baseline cystic fibrosis-derived MSSA isolates, tested with various β-lactam antibiotics at standard (SI: 10^5^ CFU/mL) and at high (HI: 10^7^ CFU/mL) inoculum

Antibiotic:	MIC_50_ (µg/mL)		MIC_90_ (µg/mL)		% IE	% pIE
	SI	HI	SI	HI		
Cefazolin	0.5	1	1	4	25.0 (*n* = 24)	0
Cefepime	4	8	8	8	1.0 (*n* = 1)	2.1 (*n* = 2)
Meropenem	0.25	0.25	0.5	0.5	0	0
Ceftazidime	16	16	32	32	5.2 (*n* = 5)	4.2 (*n* = 4)
Piperacillin-Tazobactam	2	4	8	≥128	34.4 (*n* = 33)	31.3 (*n* = 30)
Cloxacillin	0.5	1	1	2	13.5 (*n* = 13)	2.1 (*n* = 2)

**Fig 1:**
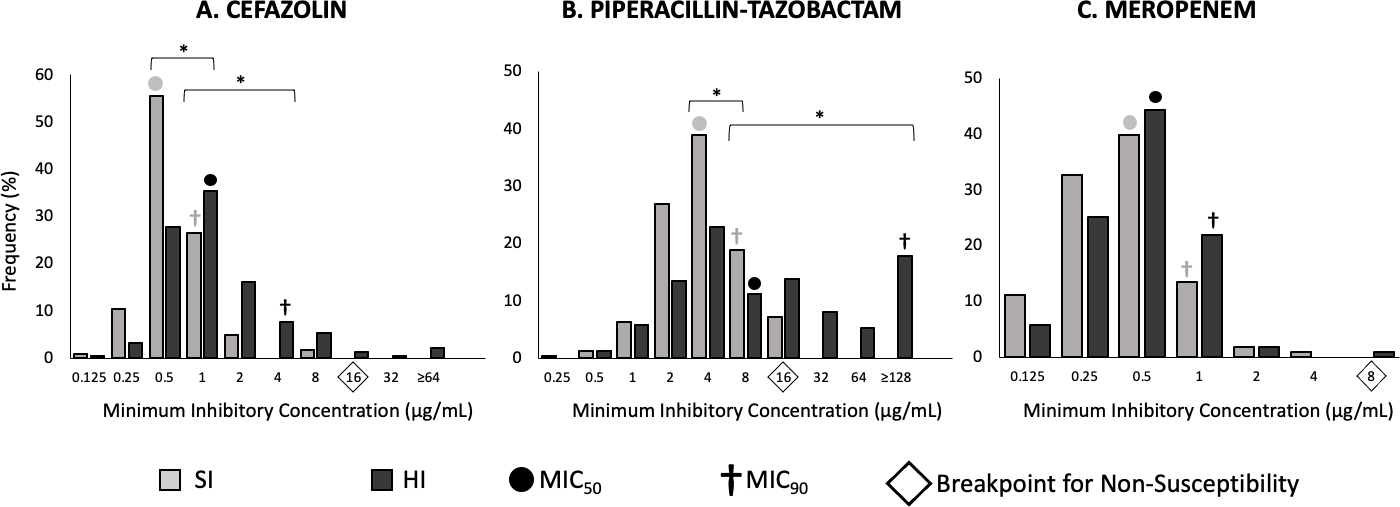
Distribution of minimum inhibitory concentrations (MICs) of 223 CF-derived MSSA isolates tested with (A) cefazolin, (B) piperacillin-tazobactam, and (C) meropenem, at standard (SI; grey) and high (HI; black) inoculum. CLSI breakpoints for non-susceptibility are noted for each antibiotic on respective panels by a diamond. Panel B shows piperacillin MIC as a varying concentration, but tazobactam was kept at a constant 4 µg/mL. Statistical significance (*t*-test) is shown between the MIC_50_ and MIC_90_ of isolates tested at standard versus high inoculum with cefazolin and piperacillin-tazobactam (*P < 0.0001).

The remaining yearly isolates (*n* = 127) were tested for susceptibility to cefazolin and piperacillin-tazobactam, owing to higher IE prevalence with these drugs, while using meropenem as a negative control. IEs were again most common with piperacillin-tazobactam; 42.6% (95 from a total 223), while 38.6% (86/223) showed pIE. Cefazolin IE prevalence was 22.0% (49/223), and pIE 3.6% (8/223). Consistently, meropenem showed no IEs. Isolates with the cefazolin or piperacillin-tazobactam IE were more likely to also display the corresponding pIE (cefazolin: RR = 0.81, CI: 0.76–0.86, *P* < 0.0001, piperacillin-tazobactam: RR = 0.76, CI:0.68–0.85, *P* < 0.0001). There was an evident rightward shift in the MIC of all three antibiotics at HI relative to SI (Fig. 1).

### Molecular epidemiology of blaZ

**Fig 2:**
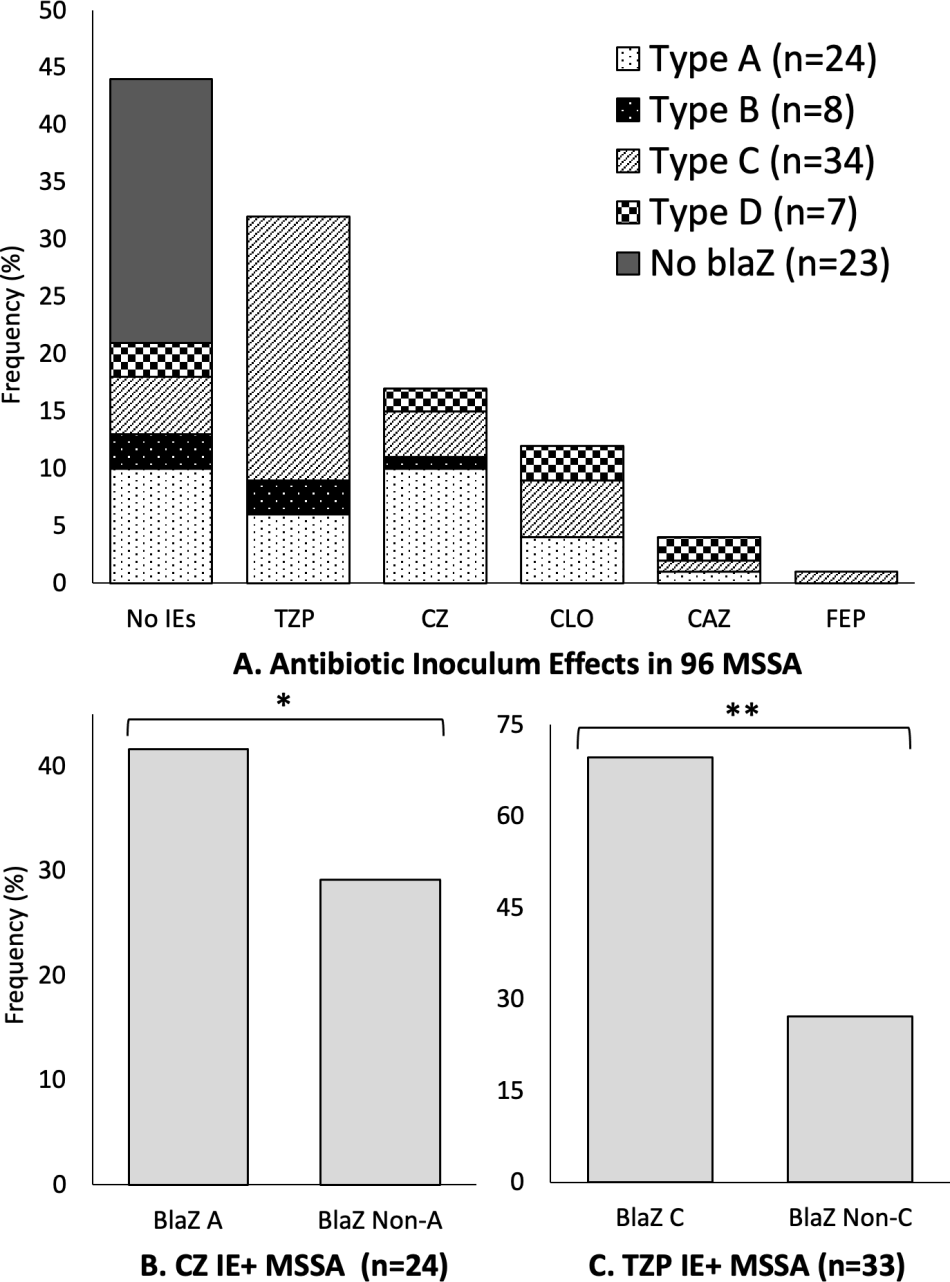
(A) Distribution of *blaZ* genotypes across 96 MSSA isolates as a function of the occurrence of inoculum effects (IE) against relevant β-lactam antibiotics. (B) The cefazolin (CZ) IE is associated with type A *blaZ* (*P = 0.0011), and (C) the piperacillin-tazobactam (TZP) IE is associated with type C *blaZ* (**P < 0.0001). Associations with *blaZ* were not noticed for IEs in cloxacillin (CLO), ceftazidime (CAZ), or cefepime (FEP).

Initial isolates (*n* = 96) were subsequently assessed for presence and allele-type of *blaZ*. Seventy-three (76.0%) were *blaZ* positive, with *blaZ* C being most common (Fig. 2). Type C *blaZ* associated with isolates exhibiting the piperacillin-tazobactam IE and pIE (OR = 14.1, 95% CI:4.5–45.5, *P* < 0.0001 and OR = 4.6, 95% CI:1.6–13.5, *P* = 0.0014, respectively) and *blaZ* A associated with isolates having cefazolin IE (OR = 6.6, 95% CI:1.9–23.9, *P* = 0.0011) (Table E6). Isolates lacking *blaZ* were less likely to have IEs overall (data not shown).

We compared our data with that of three large recent non-CF-derived MSSA studies (USA (15), Korea (18), and Latin America (20) involving 1,212 MSSA, using pooled published *blaZ* frequencies. No difference was observed in overall *blaZ* prevalence, however, non-CF-MSSA had a slightly higher proportion of *blaZ* B (*Z* = 2.97), and a lower proportion of *blaZ* D (*Z* = −5.87). These trends did not differ when the USA study alone was considered (*Z* = −3.55, *P* = 0.0004) (Table 2). We also performed an *in-silico* comparison of publicly available genomes of MSSA derived from pwCF. Three studies, two from the USA (34, 35) and one from Italy (36), met criteria. Together, 573 MSSA genomes were assessed, with 365 genomes being *blaZ*+ (63.7%). Nine were excluded; fragmented genome (*n* = 4), multiple *blaZ* alleles (*n* = 4), both fragmented and unassignable (*n* = 1). Overall, CF-published genomes had a slightly lower *blaZ* prevalence compared to our cohort (*Z* = −2.63), owing to decreased *blaZ* D (*Z* = −3.61). Prevalence of types A, B, and C did not differ (Table 2).

**TABLE 2:** Comparison of *blaZ* genotypes between our single-center study of 96 MSSA-derived *blaZ* genes and 573 pooled public *blaZ* genes obtained from globally derived CF MSSA genomes (34–36), as well as 1,212 pooled reported *blaZ* genotypes from globally derived bacteremia MSSA (15, 18, 20)^*a*^

blaZ genotype	Current study (%, *n* = 96)	Published MSSA genomes for comparison			
		blaZ reported in CF airways (%, *n* = 573)	*P*	blaZ reported in bacteremia (%, *n* = 1,212)	*P*
% blaZ Gene present	76.0 (*n* = 73)	62.1 (*n* = 356)	0.0086	79.7 (*n* = 966)	0.3953
blaZ Type:					
A	25.0 (*n* = 24)	18.0 (*n* = 103)	0.1067	22.6 (*n* = 274)	0.5892
B	8.3 (*n* = 8)	13.3 (*n* = 76)	0.1716	21.0 (*n* = 254)	0.0030
C	35.4 (*n* = 34)	29.5 (*n* = 169)	0.2446	35.5 (*n* = 430)	0.9920
D	7.3 (*n* = 7)	1.4 (*n* = 8)	0.0003	0.66 (*n* = 8)	<0.0001

^*a*^ Statistical significance is noted, and bolded when relevant. Genotype prevalences for *blaZ* are reported as proportions from respective total number of isolates.

### The inoculum effect as a strain-specific phenomenon

To establish if IE is intrinsic to individual strains, we examined strain types (MLST) amongst our total 223 MSSA. The most common strain types corresponded to ST-5 (18.4%), ST-15 (15.2%), and ST-30 (14.3%) (Table E7). ST-30 isolates were more likely to have *blaZ A* (OR = 5.5, 95% CI: 1.4–21.8, *P* = 0.0057).

**Fig 3:**
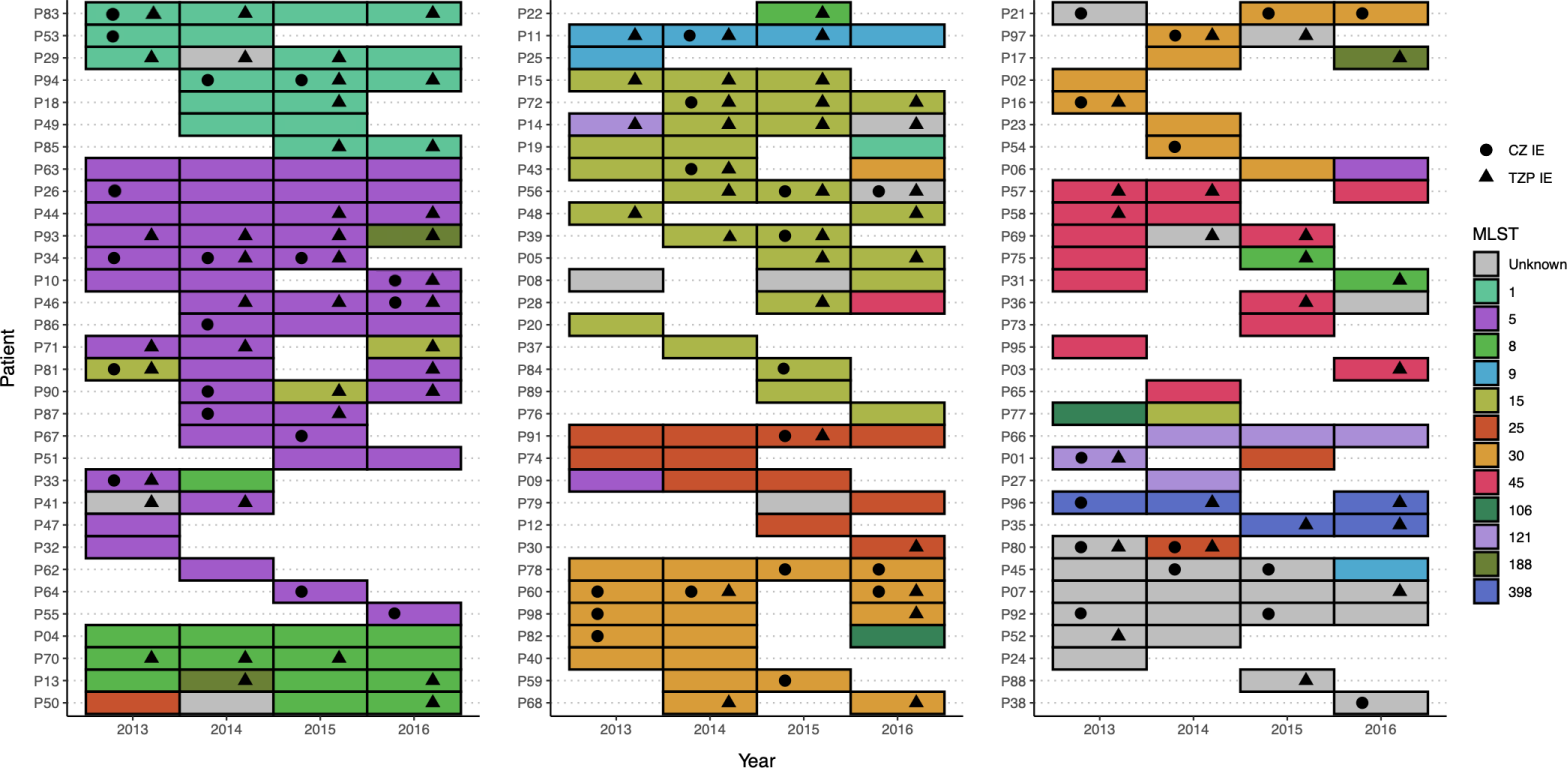
Multilocus sequence types (MLSTs), cefazolin (CZ), and piperacillin-tazobactam (TZP) inoculum effect phenotypes amongst 223 MSSA strains, derived from 96 individuals with CF over the course of four years (2013–2016). The TZP inoculum effect was found to be relatively consistent over time, where the re-occurrence of the TZP IE was common in isolates of the same strain type previously having the TZP IE. All three isolates of patient 94 were identified as ST-97, though clustered with ST-1, and were therefore noted as such.

Fifty-eight percent (56/96) of pwCF had an ST at baseline that reappeared at least once during follow-up. These STs were most commonly ST-5 (23.2%), ST-15 (16.1%), ST-30 (14.3%), and ST-1 (10.7%). Eighteen of the 56 (32.1%) had isolates that showed piperacillin-tazobactam IE at entry. Such patients were more likely to have their second MSSA of the same ST also be piperacillin-tazobactam IE positive (OR = 9.6, 95% CI: 2.1–58.6, *P* < 0.001). Likewise, individuals without initial piperacillin-tazobactam IE were more likely to have their second ST-matched strain also negative (OR = 23.4, 95% CI: 4.1–228.2, *P* < 0.0001). The most common ST amongst those isolates that preserved the piperacillin-tazobactam IE was ST-15 (7/18) (Fig. 3). Such trends were not observed with cefazolin (data not shown).

### Clinical characteristic and outcomes associated with the inoculum effect

No baseline clinical characteristics were identified associating with the risk of MSSA with cefazolin or piperacillin-tazobactam IE (Table E8). Based on the retrospective nature of this study as well as the fact that IE testing was not performed in real-time (only on representative yearly isolates), we were limited in assessing the most relevant CF outcome— individual PEx events (pulmonary function recovery and reduction in MSSA bioburden). During the four years of study, 122 exacerbations were documented in 38.5% of participants, with a median incidence of 2 PEx/participant (IQR: 1–3, range 1–25). Exacerbations lasted for a median of 13 days (IQR: 12–15d), and median time between individual PEx events was 352 d (IQR: 107–634 d). Twenty-four exacerbations (19.7%) were treated with cefazolin in 12 pwCF and four (3.3%) were treated with piperacillin-tazobactam in four pwCF. Owing to these limitations, we primarily focused on exploring long-term outcomes. Our primary outcome—rate of lung function decline over four years, did not differ based on either cefazolin or piperacillin-tazobactam IE. One patient died (end-stage lung disease), and one was transplanted, independent of IE.

The rate of BMI decline was greater for patients having at least one cefazolin or piperacillin-tazobactam IE +strain (6.1 × 10^−2^ versus 0.2 kg/m^2^/year, and −7.6 × 10^−2^ versus 0.2 kg/m^2^/year, respectively) throughout the study duration. No differences in BMI change were observed between those treated with cefazolin, or piperacillin-tazobactam for their PEx versus not (data not shown). However, patients having at least one piperacillin-tazobactam IE positive strain were more likely to have *P. aeruginosa* isolated from their sputum during their time in the cohort (OR = 3.6, 95% CI: 1.3–10.5), though this was not noticed with cefazolin (Table 3). *P. aeruginosa* density was also greater amongst those with ≥1 PEx throughout the study (10^6^, IQR: 10^5^–10^6^, versus 10^5^, IQR: 10^4^–10^6^ CFU/mL, *P* = 0.0068). *P. aeruginosa* density was not associated with an increased MSSA density amongst first isolates (Spearman *ρ* = −0.2379, *P* = 0.0196).

**TABLE 3:** Longitudinal clinical characteristics of patients with/without inoculum effect-relevant MSSA strains (2013–2016) for cefazolin and piperacillin-tazobactam^*c*^

Clinical outcome	Cefazolin			Piperacillin-tazobactam		
	IE present (*n* = 36)	IE absent (*n* = 60)	*P*	IE present (*n* = 44)	IE absent (*n* = 52)	*P*
Median decline in annual FEV_1_ (IQR) (% predicted/year) (*n* = 90)	−1.5 (−3.4–0.5)	−0.9 (−3.4–0.2)	0.9385	−1.6 (−3.3–-0.1)	−0.6 (−3.4–1.5)	0.2010
Pulmonary exacerbations during study^*a*^ (% individuals) (*n* = 96):						
No exacerbations (*n* = 59)	69.4 (*n* = 25)	56.7 (*n* = 34)	0.1518	61.4 (*n* = 27)	61.5 (*n* = 32)	0.5759
1–5 exacerbations (*n* = 32)	27.8 (*n* = 10)	36.7 (*n* = 22)	0.2525	36.4 (*n* = 16)	30.8 (*n* = 16)	0.3581
>5 exacerbations (*n* = 5)	2.8 (*n* = 1)	6.7 (*n* = 4)	0.3766	2.3 (*n* = 1)	7.7 (*n* = 4)	0.2374
Median annual BMI change (IQR) (kg/m^2^/year) (*n* = 87)	6.1 × 10^−2^ (−0.5–0.1)	0.2 (−0.2–0.7)	0.0361	−7.6 × 10^−2^ (−0.3–0.2)	0.2 (−0.1–0.7)	0.0249
Recovery of *P. aeruginosa* from sputum throughout study duration^*b*^ (% individuals) (*n* = 65)	61.1 (*n* = 22)	71.7 (*n* = 43)	0.1985	81.8 (*n* = 36)	55.8 (*n* = 29)	0.0057

^*a*^ Stratification of exacerbation frequency was based on observed trends in annual frequencies, where 1–5 exacerbations were indicative of frequencies within, and above average, while ≥ 5 frequencies were considered substantially higher than average.

^*b*^ *P. aeruginosa* was collected as mucoid or non-mucoid, and intermittent or chronic (≥50% of sputum culture during the study period), where values in the table are representative of the prevalence of any *P. aeruginosa* at any time during the study.

^*c*^ Analyses were performed by considering patients who had no IEs throughout the duration of the study, versus those with at least one IE + MSSA strain between 2013 and 2016. Not all patients had all clinical parameters available due to limitations in their clinical record, therefore, sample sizes for each clinical feature are noted.

## DISCUSSION

Despite its emergence as the most prevalent CF pathogen, there has been a relative dearth of studies on the pathobiology of MSSA. Given the rapidly evolving landscape of infection management in CF, broader assessments of the impact of MSSA and its relevant phenotypes are key to better understanding and managing infections in pwCF. IEs have not previously been explored in CF, given that clinical laboratories perform antibacterial susceptibility testing at concentrations below those relevant for IEs to become apparent (37). However, when a pwCF infected with an MSSA strain displaying the IE for a specific agent is treated with that density-sensitive agent, there exists the potential for reduced bacterial killing during pulmonary exacerbations, and, therefore, the potential for diminished lung function recovery. As pulmonary exacerbations are a primary driver of long-term disease progression in individuals with CF—optimizing their treatments will be key (38). Given the increased mortality observed in individuals experiencing MSSA bacteremia while treated with IE-sensitive agents (16), and the risk of treatment failure described in bacteremic patients with a pneumonic focus of infection (ex: high bioburden infections) (11), this first study of IEs in CF is timely.

The IE has been extensively studied in the context of bacteremia (6, 8–20) where the potent but narrow-spectrum drug, cefazolin, has been the primary agent assessed. However, as CF airway infections are frequently polymicrobial, patients are often treated with antimicrobials that cover both MSSA and Gram-negative pathogens (classically anti-pseudomonals). Indeed, in the recent STOP study (982 pwCF), cefazolin was used in only 1% of PEx (versus meropenem, 30%, ceftazidime, 28%, piperacillin-tazobactam, 22%, and cefepime, 20%) (39). Consequently, we sought to assess the IE with other CF-relevant β-lactam antibiotics with potent anti-staphylococcal activity—a topic scarcely studied in bacteremia.

We found that IEs were more prevalent than pIEs, encouraging us to focus most of our analyses on IEs. Similar to previous studies (9, 11, 15, 17, 40), we found that the prevalence of cefazolin IEs was 22.0%, yet much higher with piperacillin-tazobactam (42.6%) (18, 19). Also noted in previous work, the prevalence of IEs was low with ceftazidime (5.2%), cefepime (1.0%) (17, 18), and absent with meropenem (17, 18). The isoxazolyl penicillin, cloxacillin, was found to have limited IEs and pIEs (13.5% and 2.1%, respectively). The high prevalence of the piperacillin-tazobactam IE (42.6%) and pIE (38.6%) is of particular interest, given its use in CF as a broad-spectrum agent with concurrent anti-staphylococcal and anti-pseudomonal activity (39, 41). Although sputum MSSA density was higher in pwCF with persistent MSSA, the IE was not more common in this subgroup.

We observed that *blaZ* allele type is associated with the IE phenotype. While not all previous studies saw strict associations between *blaZ* and the IE, many works noted a high prevalence of *blaZ* A with cefazolin IE-positive MSSA (6, 9–14, 16, 20), while one observed a high prevalence of *blaZ* C with the piperacillin-tazobactam IE (18). Such associations imply a favorable structural interaction of BlaZ and the target β-lactam manifesting in greater drug hydrolysis (18). We noted a significant association between MSSA ST-30 and *blaZ* A, implying the potential role of genotype in predicting IE. Our findings align with previous studies, where associations between ST-30 MSSA and the cefazolin IE were supported (10, 12). This is relevant given the increasing incidence of ST-30 infections over time (42).

A novel observation made possible by chronic infections in CF (not previously assessed given the transient nature of bacteremia) was confirmation of IE persistence over time within serial isolates from patients infected with the same strain. Amongst pwCF whose strains were preserved, isolates with initial piperacillin-tazobactam IE were nearly 10X more likely to retain this phenotype. Such findings further suggest a strain-dependent nature underlying the IE. Persistence of the cefazolin IE over time was not confirmed (likely limited by lower overall prevalence), though associations with *blaZ* A were confirmed. Similarly, our previous study involving non-CF bronchiectasis-derived MSSA confirmed associations between cefazolin IE and ST-30, while the piperacillin-tazobactam IE associated with ST-15 (43)— further supporting the likelihood of IEs being strain-specific.

To increase the generalizability of our observations from this single-center cohort study, we compared the *blaZ* allelic distributions in our cohort to published studies involving general non-CF and CF-derived MSSA from other parts of the world. Relative to non-CF cohorts, only minor differences in *blaZ* B and D were observed. As neither of these are associated with cefazolin nor piperacillin-tazobactam IE, these differences are thought to be negligible. Overall, we demonstrated only modest differences between ours, and CF cohorts from the USA (34, 35) and Italy (36), increasing generalizability of this work.

No baseline clinical characteristics are associated with the IE in our population, so we cannot suggest demographic features that could prompt increased consideration of this phenotype. While we did not observe accelerated lung function decline between patients based on either cefazolin or piperacillin-tazobactam IE, we did note a small but significant decrease in BMI over time amongst pwCF having ≥1 cefazolin or piperacillin-tazobactam IE positive MSSA. Furthermore, those with piperacillin-tazobactam IE +MSSA were also more likely to have *P. aeruginosa* recovered in their sputum throughout the study where *P. aeruginosa* density was higher amongst those with ≥1 PEx event.

Owing to limitations in study design (ex: annual screening for IE/pIE as opposed to encounter-specific) and modest number of exacerbations experienced in this cohort, we did not attempt an exploratory analysis including PEx outcomes. To best assess the impact of IE/pIE on CF-related outcomes, a properly powered prospective pulmonary exacerbation treatment study is required. Such a study could assess for differential bacterial killing and lung function recovery in the context of PEx based on whether an individual was treated with an antibiotic to which their MSSA had inoculum-related reduced susceptibilities. The importance of study design is well-illustrated by early work on MRSA. Initial investigations to assess for impacts of MRSA on CF outcomes failed to observe differences in the rate of FEV_1_ decline in adults relative to uninfected controls (44), while larger cohorts later confirmed the now well-established differences in survival (4). Subsequent cohort studies confirmed differences in baseline FEV_1_ as a marker of disease severity (45), and greater requirements for hospitalization, and antibiotic treatments (46).

With the increasing prevalence of MSSA and decreasing prevalence of *P. aeruginosa* (1), studies assessing cefazolin will increasingly be possible and relevant. Our center previously avoided piperacillin-tazobactam use in pwCF based on historically higher rates of allergy induction with older preparations of more immunogenic ureidopenicillins relative to other β-lactams (47–49) and other associated adverse events including hypersensitivities (50), cytopenia (51), hemolytic anemia (52), and increased risk of nephrotoxicity (53) compared to alternate antipseudomonal β-lactams.

The significance of higher recovery rates of *P. aeruginosa* amongst those with MSSA with piperacillin-tazobactam IE is unknown, but similar to what has been observed with MRSA (44). While our single-center study is underpowered to detect outcome differences relative to a registry-based study, IE has not been collected in any CF administrative databases, so large-scale examinations are currently impossible. Furthermore, our study focused on adults—a group less likely to exhibit *S. aureus*-related lung function decline compared to pediatric cohorts (44). Indeed, reduced baseline lung function, disproportionate rates of lung function decline (5), and increased frequency of PEx have been noted in pediatric populations infected with SCV phenotypes (5).

Significant limitations of our biobank-based study warrant discussion. While this study involves all participants attending the only CF clinic in Southern Alberta, we did attempt to increase the generalizability by establishing that *blaZ* allelic distribution in our cohort was not markedly different than other CF and bacteremic populations throughout the world. Though we confirmed an association between *blaZ* genotype and occurrence of the IE/pIE, other genetic loci including *agr* (linked to IE/pIE in other works (16, 19, 24)) were not assessed—potentially explaining the imperfect relationships observed herein. While we did include a wide cadre of anti-Staphylococcal and more broad-spectrum CF agents, our analyses were limited to the most relevant drugs, and we did not explore this phenomenon in newer β-lactams and antibiotics of other classes.

### Conclusions

In this first study of the IE/pIE in CF, we have established that MSSA within CF airways exists at high-density, and that many strains display the phenotype of inoculum-related reduced susceptibility—particularly to cefazolin and piperacillin-tazobactam. As *S. aureus* is now the most prevalent CF pathogen and other phenotypes have significant impacts on CF-outcomes, our study serves as a call-to-action to begin further CF-IE/pIE investigations, given their prevalence.

## SUPPLEMENTAL MATERIAL

Supplemental file 1 aac.00136-23-s0001.docx Supplemental text and tables.

Supplemental file 2 aac.00136-23-s0002.pdf Supplemental Figure E1.

ASM does not own the copyrights to Supplemental Material that may be linked to, or accessed through, an article. The authors have granted ASM a non-exclusive, world-wide license to publish the Supplemental Material files. Please contact the corresponding author directly for reuse.
