# Supplementary material for: Epidemiology and impact of methicillin-sensitive Staphylococcus aureus with β-lactam antibiotic inoculum effects in adults with cystic fibrosis
Source: Antimicrob Agents Chemother. 2023 Nov 15;67(12):e00136-23. doi: 10.1128/aac.00136-23 (PMC10720481; doi:10.1128/aac.00136-23)
Supplement: Supplemental file 1 — Supplemental text and tables. [file aac.00136-23-s0001.docx]

**Online Supplement**

**Epidemiology and impact of methicillin-sensitive *Staphylococcus aureus* with β-lactam antibiotic inoculum effects in adults with cystic fibrosis**

Svishchuk, J.^1^ and Ebbert, K.^2^, Waddell, B.^1^, Izydorczyk, C.^1^., Acosta, N.^1^, Somayaji, R.^1,3^, Rabin, H.R.^1^, Bjornson, C.L.^2^, Lisboa, L.^4^, Gregson, D.B.^3,4^, Conly, J.M.^1,3,4^, Surette, M.G^5^, and Parkins, M.D*^1,3^

^1^Department of Microbiology, Immunology, and Infectious Diseases, University of Calgary, Calgary, AB

^2^Department of Pediatrics, University of Calgary, Calgary, AB

^3^Department of Medicine, University of Calgary and Alberta Health Services, Calgary, AB

^4^Department of Pathology and Laboratory Medicine, University of Calgary and Alberta Health Services, Calgary, AB

^5^Department of Biochemistry and Biomedical Sciences, McMaster University, Hamilton, ON

***Corresponding Author:**

Michael D. Parkins, MD MSc FRCPC

Professor, Departments of Medicine and Microbiology, Immunology & Infectious Diseases University of Calgary

Medical Director Calgary Adult CF Clinic, Alberta Health Services

Section Chief, Infectious Diseases, Alberta Health Services

Postal address: 3330 Hospital Drive, NW, Calgary, Alberta, Canada, T2N 4N1

E-mail: mdparkin@ucalgary.ca

Phone: 403-220-5951, Fax: 403-270-2772.

**Methods:**

***Sputum Collection and Processing***

Sputum samples were submitted regularly by each participant as part of routine care. Standard of care included collection of sputum for microbial analysis on a quarterly basis and with every pulmonary exacerbation (PEx). Sputum samples were cultured in real-time using the same protocol through the studies duration. Upon receipt, sputum samples were mixed with equal volumes of dithiothreitol/“Sputolysin” (EMD Millipore, MA, USA), and serially diluted in sterile saline to 1:10^3^ and 1:10^5^. Resulting dilutions were plated on Columbia Blood Agar, MacConkey Agar, Mannitol Salt Agar, Chocolate Agar, and *Burkholderia cepacia* selective agar, and colonies were counted after 48 hours of incubation at 37°C. Colony count assessments were done independently on all representative morphotypes of each identified species and following Table E1. *S. aureus* isolates were confirmed as MSSA or MRSA using standard approaches, and representative isolates from all individual morphotypes were frozen in glycerol or skim milk in the Calgary Adult Cystic Fibrosis Clinic (CACFC) biobank. Any small colony variants (SCV) were independently assessed if identified. Only MSSA isolates were assessed as part of this work.

**Table E1: Colony count assessment on cystic fibrosis sputum samples for individual morphotypes.**

| **Dilution** | **Number of Colonies on Plate** | | |
| --- | --- | --- | --- |
|  | 3-9 | 10-99 | ≥100 |
|  | **Quantification (CFU/mL)** | | |
| **10^-3^** | 10^3^ | 10^4^ | ≥10^7^ |
| **10^-5^** | 10^5^ | 10^6^ | ≥10^7^ |

***High Inoculum Testing of CF MSSA Isolates***

All MSSA isolates were retrospectively recovered from our biobank and grown on Bacto^TM^ tryptic soy agar (TSA; Becton, Dickinson and Company, MD, USA) overnight at 37°C, and suspensions were made in 0.85% saline solution. High inoculum (HI) working suspensions of 5 x 10^8^CFU/mL were created using a 1 MacFarland turbidity standard (Remel^TM^, ThermoScientific, MA, USA), and were compared against a MacFarland equivalence turbidity card by two researchers, while every 10^th^ suspension was verified spectrophotometrically (at 600nm) maintaining absorbance between 0.25 and 0.30 as per the 2009 CLSI guidelines^1^. SI working suspensions (at 5 x 10^6^ CFU/mL) were generated by creating 1:10^2^ dilutions of HI working suspensions in 0.85% saline.

All antibiotics tested (purchased from Sigma-Aldrich, MO, USA) were selected based on their clinical significance – either for optimally managing MSSA infections, or for their frequent use in managing CF airways infections. Antibiotics were prepared in stock and serially diluted in BBL^TM^ cation-adjusted Mueller-Hinton II broth (Becton, Dickinson, and Company, MD, USA) in 96-well plates as previously described^2^. Each MSSA isolate was then inoculated in these 96-well plates at SI (5 x 10^6^ CFU/mL) and at HI (5 x 10^7^ CFU/mL) by pipetting 10µL volumes of respective working suspensions, leaving row 12 without bacteria to use as a blank. Row 11 was used as a growth control and contained no antibiotics. Plates were incubated overnight at 37°C, and bacterial growth was determined spectrophotometrically at 600nm via a Wallac Victor^2^ 1420 multilabel plate counter. Previously, it was established that absorbances of ≥0.100 were colourimetrically indicative of MSSA growth^2^ using the alamarBlue^TM^ HS cell viability reagent (Invitrogen, OR, USA) (data not shown), thus, this cut-off was used to establish minimum inhibitory concentrations (MICs) for each isolate per each antibiotic. Initial suspension concentrations were verified again by plate counting following overnight incubation; 10µL of broth from the growth control was transferred into 90µL of 0.85% saline in a separate 96-well plate, and serially diluted 5X in saline, then spotted onto TSY agar at 10µL volumes. Agar plates were incubated overnight at 37°C, and colonies were counted. Accurate suspension concentrations yielded 2-8 colonies per spot with bacterial inocula of 10^5^CFU/mL and 10^7^CFU/mL (SI and HI, respectively). If colony counts of an isolate failed to fall within this range, the data was discarded, and the isolate was re-tested.

Control MSSA strains ATCC® 25923^TM^ (no *blaZ* gene) and ATCC® 29213^TM^ (a weak BlaZ producer) were used to ensure accurate antibiotic dilutions. Minimal inhibition concentrations (MICs) were reported as MIC_50_ and MIC_90_ values – the lowest concentration of antibiotic at which 50% and 90% of isolates’ growth were inhibited, respectively. MICs at SI and HI were used to define inoculum effects (as outlined in the main Methods), and 50^th^ and 90^th^ percentiles of MICs were used for comparative analyses (Table 1 and Figure 1). Isolates were defined as **“non-susceptible”** to a given antibiotic if the MIC for a given isolate against an antibiotic was classified as “intermediate/I” or “resistant/R” in the 2009 CLSI guidelines for antimicrobial susceptibility via broth microdilution^1^ (Table E2).

A select number of isolates showed particular difficulty resuspending in saline, and are marked with a † in Table E7. These isolates showed higher SI than HI MIC for certain antibiotics. Upon observing this trend (generally a sticky phenotype), isolates marked with a † were re-tested in duplicate twice, on separate days, with newly-made antibiotic stocks. All MICs determined were within one dilution from original MICs (data not shown), and original MICs were therefore reported in Table E7.

**Table E2**: **Protocols followed for antibiotic preparation.** The table indicates what solvent each antibiotic stock was prepared in, the concentrations spanned on each 96-well plate for MIC testing, breakpoints for antibiotic susceptibility, intermediacy, and resistance, as well as the MIC range for the control strain (ATCC® 29213^TM^) at standard inoculum (SI). These protocols were adapted from the 2009 CLSI guidelines for antimicrobial susceptibility testing^1^.

| **Antibiotic** | **Stock Solvent** | **Concentration Range (µg/mL)** | **MIC Breakpoints (µg/mL)** | **MIC Range for ATCC® 29213^TM^ at SI (µg/mL)** |
| --- | --- | --- | --- | --- |
| **Cefazolin (CZ)** | 0.1M Phosphate Buffer, pH 6.0 | 0.125 - 64 | S ≤ 8  I = 16  R ≥ 32 | 0.25 - 1 |
| **Cefepime (FEP)** | 0.1M Phosphate Buffer, pH 6.0 | 0.125 - 64 | S ≤ 8  I = 16  R ≥ 32 | 1 - 4 |
| **Meropenem (MEM)** | DDW | 0.03 - 16 | S ≤ 4  I = 8  R ≥ 16 | 0.03 – 0.12 |
| **Ceftazidime (CAZ)** | DDW + 10% Na_2_CO_3_ | 0.125 - 64 | S ≤ 8  I = 16  R ≥ 32 | 4 - 16 |
| ***Piperacillin-Tazobactam (TZP)** | DDW | 0.25/4 - 128/4 | S ≤ 8/4  R ≥ 16/4 | 0.25/4 – 2/4 |
| **Cloxacillin (CLO)** | DDW | 0.0625 - 32 | S ≤ 2  R ≥ 4 | 0.125 – 0.5 |

DDW: Double-distilled water

S: Sensitive, I: Intermediate, R: Resistant

*Concentrations for TZP susceptibility are expressed as piperacillin/tazobactam.

***Gene Typing of Staphylococcal blaZ from* CACFC cohort**

The first isolate of each patient (n=96) was amplified using a standard polymerase chain reaction (PCR) with Phusion^TM^ High-Fidelity DNA Polymerase (ThermoScientific, MA, USA) as outlined in Table E3. Amplified samples were run on a 1% agarose gel to assess for *blaZ* presence and sent for Sanger sequencing to the University of Calgary’s local sequencing facility. Isolates negative for the *blaZ* gene were confirmed as negative by re-amplifying them with an alternative primer set, designed on Primer BLAST.

**Table E3:** **Standard PCR conditions used to identify and amplify the *blaZ* gene in 96 MSSA isolates.** Two sets of primers were used in the analysis; one for the initial detection of the *blaZ* gene for all 96 MSSA isolates (obtained from Wang et al.^3^), and another for confirmation of *blaZ* absence in those samples that did not show a band for *blaZ* at electrophoresis (designed on BLAST).

| **PCR Parameter** | **PCR Setting Used (50µL)** | **Primers for *blaZ* amplification for all isolates (n=96)** | **Primers for verification of *blaZ*-negative strains (n=23)** |
| --- | --- | --- | --- |
| **Denaturation** | 95°C  2 minutes | **Forward:**  5’-ATT TTG AAA AAG TTA ATA TTT TTA ATT G-3’ | **Forward:**  5’-CAA CGT CTA AAA GAA CTA GG-3’ |
| **Annealing** | 94°C  30 seconds |  |  |
| **Elongation** | 53.8°C  1 minute | **Reverse:**  5’-CAT TAC ACT CTT GGC GGT TTC-3’ | **Reverse:**  5’-CCT TCA TTA CAC TCT TGG-3’ |
| **Number of Cycles** | 35 |  |  |
| **Infinite Hold** | 12°C |  |  |

Sequences obtained from isolates positive for the *blaZ* gene were manually converted into FASTA format and uploaded to the Comprehensive Antibiotic Resistance Database (CARD, version 3.1.4) Resistance Gene Identifier (RGI, version 5.2.0), available online (https://card.mcmaster.ca/analyze/rgi). CARD-derived predicted amino acid sequences were input into BLASTp and aligned against the PC1 *blaZ* type A protein sequence (reference: WP_000733283.1). MSSA isolates were manually defined as types A, B, C, or D according to single nucleotide polymorphisms (SNPs) indicated in Voladri and Kernodle^4^, specifically by assessing amino acid residues 128 and 216.

***Screening for blaZ in Published non-CF MSSA Genomes***

The majority of work on inoculum effects in MSSA has been specific to cefazolin, particularly in the context of bacteremia. For our analyses, we selected studies that reported *blaZ* allele frequencies amongst a set of MSSA screened for β-lactam inoculum effects, derived from any non-CF source, published between January 2017-January 2022. To maximize the generalizability of our work, we included only studies that were conducted in individual countries outside of Canada and contained ≥200 individual patient-derived MSSA isolates. For inclusion, studies needed to have performed *blaZ* typing via Voladri and Kernodle, or an equivalent approach – specifically using amino acids positions 128 and 216 in the *blaZ* gene to differentiate between types. Studies that had less than 200 MSSA isolates, did not perform/report *blaZ* allele frequency analyses, used an alternate approach to subtype *blaZ*, or were published before 2017 were excluded. Where two or more studies from the same country met our criteria, the study with the greater number of isolates was selected.

Three studies were identified that met our inclusion/exclusion criteria^3,5,6^. The study from Wang et al, 2018^3^ included isolates collected from four Chicago-area hospitals obtained from patient’s bloodstream infections, as well as wound abscesses, tracheal swabs, sputum, and other bodily fluids (n=269 MSSA). The study from Song et al, 2019 included ten Korean hospitals and contained only bacteremic isolates (n=302 MSSA). The final study by Carvajal et al, 2020^6^ included bacteremic isolates collected from nine hospitals across Latin America (n=690 MSSA).

***Screening for blaZ in Published CF-Derived MSSA Genomes***

Published data from CF studies on *blaZ* frequency do not exist. Likewise, studies assessing the genomic content of CF-derived *S. aureus* are sparse, with relatively low cohort sizes. Accordingly, we sought out studies where whole genome sequence data on *S. aureus* (MSSA and MRSA) derived from individuals with CF was available. Inclusion criteria necessitated being published in the between January 2017-January 2022, and containing ≥50, but ≤500 isolates from different individuals (for feasibility of data handling). Once again, we selected only studies published from outside of Canada. Studies including only MRSA isolates were excluded.

Three published studies (NCBI BioProject IDs PRJNA380429^7^, PRJNA480016^8^, and PRJNA400143^9^) meeting these criteria*. S. aureus* were analyzed for the presence/type of *blaZ* as a comparator cohort to our local CACFC cohort. Sequencing reads (fastq files) for each isolate were downloaded from ENA for samples from PRJNA48016 and PRJNA400143 using enaDataGet (<https://github.com/enasequence/enaBrowserTools>, v1.5.3), while *de novo* assemblies were downloaded directly from NCBI for isolates from PRJNA380429. Sequencing quality of downloaded sequencing reads was analyzed with FastQC (<https://www.bioinformatics.babraham.ac.uk/projects/fastqc/>, v0.11.9), and read trimming was performed with Trimmomatic^10^ (v0.39) with the following options: ILLUMINACLIP:<path/to/TruSeq3-PE-2.fa>:2:30:10:8:true, CROP:300, SLIDINGWINDOW:4:20, and MINLEN:31. Sequencing reads were then assembled (separately) using Unicycler^11^ (v0.5.0) and SKESA^12^ (v2.4.0), both with default settings. Bakta^13^ (v1.5.1 and database version 4.0) (with default settings and the --compliant option set) was then used to annotate *de novo* assemblies from both Unicycler and SKESA, as well as to re-annotate assemblies downloaded from PRJNA380429. Annotated assemblies from all three BioProjects were then queried for the presence of *blaZ* using AMRFinderPlus^14^ (v3.10.40), the Resistance Gene Identifier (RGI) (v6.0.0) and the latest CARD database^15^ (accessed October 2022)c, and ResFinder^16^ (v4.2) (databases downloaded September 2022). The combination of Unicycler + SKESA assemblies and three antimicrobial resistance gene detection tools was used to ensure accurate identification of *blaZ* and to avoid false negative gene calls for each isolate, which were observed when only a single assembler/tool was used. Custom python scripts available at (https://github.com/cizydorczyk/Epi-of-MSSA-with-B-lactam-inoculum-effects-in-CF) were used to extract *blaZ* gene sequences from Bakta annotations and obtain the *blaZ* type^4^ from an alignment of *blaZ* sequences generated by Mafft^17^ (v7.508) with default settings.

***Pulsotyping and MLST Identification of MSSA Isolates***

*S. aureus* isolates were typed via pulsed-field gel electrophoresis (PFGE)^18^ using protocols adapted from Mulvey^19^, Struelens^20^ and PulseNet. Isolates were plated overnight on TSY agar and suspended in cell suspension buffer (CSB: 10mM Tris-HCl pH 7.2, 20mM NaCl, 50mM EDTA) to a McFarland turbidity standard of 1.5 or to an OD600 of 0.5-0.6. Two microlitres of 1mg/mL Lysostaphin was added to 150ul of each suspension. Equal amounts of melted 1% SeaKem agarose (Lonza Ca.50150) in CSB was mixed with suspensions and added to plug molds, then allowed to cool. Plugs were lysed in 500µL of cell lysis buffer (10mM Tris-HCl pH 7.2, 50mM NaCl, 50mM EDTA, 0.2% deoxycholate, 0.5% N-Laurylsarcosine) for 1.5 hours at 37^˚^C followed by the addition of 500µL of Proteinase K buffer (250mM EDTA pH 9.0, 1% N-Laurylsarcosine, 100ug/mL Proteinase K) incubated over night at 55^˚^C. Plugs were rinsed twice in 1mL of wash buffer (10mM Tris-HCl pH 7.6, 0.1mM EDTA) followed by an additional three times for 15 minutes at 37˚C. Plugs were then rinsed in 0.5X CutSmart Buffer (NEB B7204S) for 15 minutes and then digested with 40U Sma1 (NEB R0141L) in 1x CutSmart buffer at 25^˚^C for 2 hours. Plugs were washed in wash buffer three more times for 5 minutes before loading onto a 1% SeaKem Gold agarose (Lonza Ca.50150) gel in 2 litres of 0.1X TBE buffer (Invitrogen Ca.15581-044). *Salmonella braenderup* H9812 ladder digested in 40U Xba1 (NEB R0145L) was used for reference (PulseNet, Salmonella SOP). The gels were run on a Chef Mapper (BioRad) at 6 Volts, 120˚ included angle, switch times in 5s and in 35s for 19 hours in 0.1X TBE buffer at 10^˚^C. Gels were stained in GelRed (Lonza Ca.41003) and imaged on a ChemiDoc Touch (BioRad). Dendrograms were generated with BioNumerics version 7.6 (Applied Maths, Austin, TX), using the unweighted pair-group method with arithmetic mean (UPMGA) and a similarity index of ≥80%. Pulsogroups were assigned to clusters of more than 2 patients with isolates sharing > 80% identity on dendrograms (Figure E1).

Multilocus sequence typing (MLST) of representative isolates within pulsogroups was accomplished with stringMLST from whole genome sequenced isolates. Genomic DNA was isolated from overnight cultures grown on TSY agar using the Promega Wizard Genomic DNA purification kit. Libraries were prepared using Protocol 3 of the NEBNext Ultra FS II library kit (NEB E7805L) with Illumina dual index primers (NEB E7600S, E77805). Protocol 3 was used with half volumes throughout with a 3-minute fragmentation time. Illumina MiSeq V2 (2x300bp reads, 15Mrp) was performed on libraries.

Sequence quality of raw reads was analyzed using FastQC (version 0.11.9) while read trimming to remove adapters was performed on Trimmomatic (version 0.39) (option ILLUMINACLIP:/path/to/adapters/file:2:30:10:8:true, remove the 301^st^ base (option CROP:300)), trim reads from the 3’ end if the average Phred quality fell below 5 in a sliding window of 4 bp (option SLIDINGWINDOW:4:5), and remove reads shorter than 30 bp after all previous trimming steps (option MINLEN:30)^10^). Isolates were typed *in-silico* with stringMLST (version 0.6.3) using trimmed sequencing reads^21^. The *S. aureus* MLST database was downloaded using stringMLST. Default settings were used to assign sequence types.

***Clinical Characteristics and Patient Outcome Assessment***

Clinical characteristics and demographics of study participants were collected through a detailed chart audit. Factors in the study included: age, sex, CFTR genotype, percent predicted forced vital capacity (FVC) and forced expiratory volume in one second (FEV_1_), body mass index (BMI) measured as kg/m^2^, pancreatic status and CF-related comorbidities (including CF-related diabetes, CF-liver disease, allergic bronchopulmonary aspergillosis (ABPA), and CF-sinus disease), and CF therapies. Treatment with a CFTR-modulator, or participation in a CFTR-modulator trial was recorded. FVC and FEV_1_ were captured at all outpatient and inpatient encounters during which pulmonary function testing was performed. Pulmonary exacerbations were defined as those being treated with parenteral antimicrobial therapies. Concordance between local definitions of exacerbations and Fuchs has previously been established^22^.

**Results**

***MSSA Susceptibility Testing***

The median MIC_50_ for isolates tested with CZ shifted from 0.5µg/mL (IQR: 0.5-1µg/mL) at SI, to 1µg/mL (IQR: 0.5-2µg/mL) at HI (p<0.0001). Similarly, the median MIC_50_ for TZP increased from 4µg/mL (IQR: 2-4µg/mL) at SI, to 8µg/mL (IQR: 4-16µg/mL) at HI (p<0.0001). MIC_90_ likewise increased from SI to HI for both CZ (from 1µg/mL to 4µg/mL) and TZP (from 8 µg/mL to ≥128 µg/mL) (p<0.0001). No isolates showed any resistance to CZ at SI, but 7.2% (16/223) showed non-susceptibility to TZP at SI, while 4.0% (9/223) were non-susceptible to CZ at HI, and 45.3% (101/223) were non-susceptible to TZP at HI (Figure 1).

**Table E4:** **Baseline clinical characteristics at study entry of 96 persons with cystic fibrosis having at least one MSSA+ sputum sample between 2013 and 2016.**

| **Patient Characteristic** | **Prevalence in Cohort** |
| --- | --- |
| Genotype (%):  F508del homozygous  F508del heterozygous  Other | 50.0% (n=48)  34.4% (n=33)  15.6% (n=15) |
| Pancreatic Insufficient (%) | 77.1% (n=74) |
| BMI (median (IQR), kg/m^2^) | 21.7 (19.7-24.4) |
| Baseline FEV_1_ (%)  $\geq$80% predicted  60-79% predicted  40-59% predicted  <40% predicted | 32.3% (n=31)  29.2% (n=28)  22.9% (n=22)  15.6% (n=15) |
| CF Comorbidities (%):  CFRD CFLD  ABPA  CF Sinus Disease | 14.6% (n=14)  28.1% (n=27)  5.3% (n=5)  55.2% (n=53) |
| Chronic CF Therapies (%):  Chronic Oral Antibiotics  Chronic Inhaled Antibiotics**  Nebulized Dornase Alfa  Nebulized Hypertonic Saline  Azithromycin  Insulin  Enteral Nutrition  CFTR Modulator*** | 12.5% (n=12)  29.2% (n=28)  42.7% (n=41)  18.8% (n=18)  27.1% (n=26)  7.3% (n=7)  5.2% (n=5)  4.2% (n=4) |

CFRD: CF-related diabetes, CFLD: CF-related liver disease, ABPA: allergic bronchopulmonary aspergillosis.

**Inhaled antibiotics included tobramycin, aztreonam, colistin, and/or vancomycin.

***A total of 4/96 patients were on a CFTR modulator or enrolled in a CFTR modulator trial at any point throughout the duration of the study.

**Table E5: Pathogens isolated from the airways of individuals with CF having persistent MSSA during their first year in the study.** Persistent MSSA was defined as an isolate being recovered during a year in which ≥50% of that patient’s sputum cultures were MSSA+.

| **Co-Infecting Pathogens**  **(Prevalence amongst 96 pwCF)** | **Persistent MSSA (n=60)**  **(% individuals from 96)** |
| --- | --- |
| -MRSA (n=15)  -SCV *S. aureus* (n=1)  *-Pseudomonas aeruginosa* (n=65)  *-Haemophilus influenzae* (n=22)  *-Stenotrophomonas maltophilia* (n=23)  *-Streptococcus pneumoniae* (n=3)  -*Achromobacter spp* (n=8)  *-Klebsiella pneumoniae* (n=2)  *–Burkholderia* cepacian complex* (n=6)  *-Aspergillus fumigatus* (n=28)  –Non-tuberculous mycobacteria** (n=21) | 6.3 (n=6)  0 (n=0)  38.5 (n=36)  15.6 (n=15)  15.6 (n=15)  2.1 (n=2)  6.3 (n=6)  1.0 (n=1)  4.2 (n=4)  20.8 (n=20)  16.7 (n=16) |

**Burkholderia* cepacian complex included *B. cenocepacia* and *B. multivorans.*

****Non-tuberculous mycobacteria included members of the *M. avian* complex and *M. abscesses* complex.

**Table E6: Distribution of staphylococcal *blaZ* types across the first MSSA isolates obtained from each of 96 patients with cystic fibrosis, across isolates positive and negative for inoculum effects with cefazolin (CZ), cefepime (FEP), meropenem (MEM), ceftazidime (CAZ), piperacillin-tazobactam (TZP), and cloxacillin (CLO).** Statistical associations are likewise indicated, with significant parameters bolded where a Bonferroni-corrected alpha of 0.00208 was used for significance. Comparison analyses were not performed for MEM, as no IEs were identified.

| **Antibiotic/**  **Inoculum Effect (IE)** | | ***blaZ* Type** | | | |
| --- | --- | --- | --- | --- | --- |
|  |  | **A**  **(n=24)** | **B**  **(n=8)** | **C**  **(n=34)** | **D**  **(n=7)** |
| **CZ** | **IE+ (%)**  **(n=24)** | 41.7 (n=10/24) | 12.5 (n=1/8) | 11.8 (n=4/34) | 28.6 (n=2/7) |
|  | **IE- (%)**  **(n=72)** | 19.4 (n=14/72) | 8.0 (n=7/88) | 48.4 (n=30/62) | 5.6 (n=5/89) |
|  | ***P*** | **0.0011** | 0.3569 | 0.0214 | 0.5618 |
| **FEP** | **IE+ (%)**  **(n=1)** | 0 (n=0/24) | 0 (n=0/8) | 2.9 (n=1/34) | 0 (n=0/7) |
|  | **IE- (%)**  **(n=96)** | 33.3 (n=24/72) | 9.1 (n=8/88) | 53.2 (n=33/62) | 7.9 (n=7/89) |
|  | ***P*** | 0.7500 | 0.9167 | 0.3542 | 0.9271 |
| **MEM** | **IE+ (%)**  **(n=0)** | 0 (n=0/24) | 0 (n=0/8) | 0 (n=0/34) | 0 (n=0/7) |
|  | **IE- (%)**  **(n=96)** | 33.3 (n=24/72) | 9.1 (n=8/88) | 51.6 (n=32/62) | 7.9 (n=7/89) |
|  | ***P*** | N/A | N/A | N/A | N/A |
| **CAZ** | **IE+ (%)**  **(n=5)** | 4.2 (n=1/24) | 0 (n=0/8) | 2.9 (n=1/34) | 28.6 (n=2/7) |
|  | **IE- (%)**  **(n=91)** | 31.9 (n=23/72) | 9.1 (n=8/88) | 53.2 (n=33/62) | 5.6 (n=5/89) |
|  | ***P*** | 0.6329 | 0.6409 | 0.4162 | 0.0413 |
| **TZP** | **IE+ (%)**  **(n=33)** | 25.0 (n=6/24) | 37.5 (n=3/8) | 67.6 (n=23/34) | 0 (n=0) |
|  | **IE- (%)**  **(n=63)** | 25.0 (n=18/72) | 5.7 (n=5/88) | 17.7 (n=11/62) | 7.9 (n=7/89) |
|  | ***P*** | 0.1937 | 0.5626 | **<0.0001** | 0.0464 |
| **CLO** | **IE+ (%)**  **(n=13)** | 16.7 (n=4/24) | 0 (n=0/8) | 14.7 (n=5/34) | 42.9 (n=3/7) |
|  | **IE- (%)**  **(n=83)** | 27.8 (n=20/72) | 9.1 (n=8/88) | 46.8 (n=29/62) | 4.5 (n=4/89) |
|  | ***P*** | 0.4155 | 0.2975 | 0.5166 | 0.0500 |

**Table E7: Data obtained from 223 MSSA isolates (96 patients) including isolate collection date, *blaZ* type for initial isolates, multilocus sequence type (MLST), and minimum inhibitory concentration for relevant antibiotics against isolates at standard inoculum (SI) and high inoculum (HI)**. “NA” indicates that the given value is not applicable to the respective characteristic. Isolates exhibiting the inoculum effect by Definition 1 (“IE”, mid-grey), Definition 2 (“pIE”, light grey), and both (dark grey) are identified.

|  | **Isolate Characteristics** | | | | | ****Minimum Inhibitory Concentration (MIC) of MSSA to Various β-Lactam Antibiotics (µg/mL)** | | | | | | | | | | | |
| --- | --- | --- | --- | --- | --- | --- | --- | --- | --- | --- | --- | --- | --- | --- | --- | --- | --- |
| ***Patient Number** | **Patient ID** | **Isolate Number** | **Collection Date** | ***blaZ* Type** | **MLST** | **CZ SI** | **CZ HI** | **FEP SI** | **FEP HI** | **MEM SI** | **MEM HI** | **CAZ SI** | **CAZ HI** | **TZP SI** | **TZP HI** | **CLO SI** | **CLO HI** |
| **1** | **A280** | **514** | 14-Mar-13 | C | 121 | 0.5 | 1 | 8 | 8 | 0.25 | 0.25 | 32 | 32 | 4 | 16 | 1 | 1 |
|  |  | **557** | 02-Mar-15 | NA | 25 | 0.25 | 1 | NA | NA | 0.25 | 0.5 | NA | NA | 2 | 4 | NA | NA |
| **2** | **A378** | **551** | 29-May-13 | A | 30 | 0.5 | 1 | 4 | 4 | 0.25 | 0.25 | 8 | 8 | 2 | 4 | 0.5 | 1 |
| **3** | **A058** | **520** | 14-Mar-16 | C | 45 | 0.5 | 1 | 4 | 4 | 0.25 | 0.5 | 16 | 16 | 8 | ≥128 | 1 | 2 |
| **4** | **A383** | **553** | 09-Dec-13 | Absent | 8 | 0.5 | 1 | 4 | 8 | 0.25 | 0.25 | 8 | 8 | 2 | 2 | 1 | 1 |
|  |  | **555** | 05-Nov-14 | NA | 8 | 0.5 | 0.5 | NA | NA | 0.25 | 0.5 | NA | NA | 1 | 1 | NA | NA |
|  |  | **561** | 19-Oct-15 | NA | 8 | 1 | 2 | NA | NA | 0.5 | 1 | NA | NA | 2 | 2 | NA | NA |
|  |  | **563** | 12-Jul-16 | NA | 8 | 0.5 | 1 | NA | NA | 0.5 | 0.5 | NA | NA | 2 | 2 | NA | NA |
| **5** | **A379** | **558** | 02-Sep-15 | C | 15 | 0.5 | 1 | 4 | 8 | 0.25 | 0.25 | 8 | 8 | 8 | ≥128 | 1 | 1 |
|  |  | **315** | 27-Jul-16 | NA | 15 | 1 | 2 | NA | NA | 1 | 1 | NA | NA | 8 | ≥128 | NA | NA |
| **6** | **A380** | **559** | 21-Sep-15 | B | 30 | 0.25 | 0.5 | 4 | 8 | 0.125 | 0.125 | 8 | 8 | 2 | 4 | 0.25 | 0.5 |
|  |  | **562** | 26-Apr-16 | NA | 5 | 0.5 | 0.5 | NA | NA | 0.25 | 0.5 | NA | NA | 2 | 2 | NA | NA |
| **7** | **A009** | **550** | 01-Apr-13 | Absent | Unknown | 0.25 | 0.5 | 2 | 4 | 0.125 | 0.25 | 8 | 8 | 1 | 2 | 0.5 | 0.5 |
|  |  | **282** | 03-Feb-14 | NA | Unknown | 0.5 | 1 | NA | NA | 1 | 1 | NA | NA | 4 | 4 | NA | NA |
|  |  | **309** | 02-Nov-15 | NA | Unknown | 1 | 1 | NA | NA | 2 | 2 | NA | NA | 4 | 4 | NA | NA |
|  |  | **225** | 19-Sep-16 | NA | Unknown | 1 | 1 | NA | NA | 0.5 | 0.5 | NA | NA | 4 | 64 | NA | NA |
| **8** | **A055** | **295**† | 08-May-13 | Absent | Unknown | 0.25 | 0.5 | 4 | 4 | 0.5 | 0.25 | 16 | 32 | 4 | 8 | 1 | 1 |
|  |  | **560** | 21-Sep-15 | NA | Unknown | 0.5 | 0.5 | NA | NA | 0.25 | 0.5 | NA | NA | 2 | 2 | NA | NA |
|  |  | **403** | 21-Nov-16 | NA | 15 | 1 | 1 | NA | NA | 2 | 2 | NA | NA | 4 | 4 | NA | NA |
| **9** | **A381** | **12** | 16-Sep-13 | Absent | 5 | 1 | 1 | 8 | 8 | 0.5 | 1 | 4 | 4 | 2 | 2 | 1 | 1 |
|  |  | **294** | 22-Sep-14 | NA | 25 | 1 | 2 | NA | NA | 1 | 1 | NA | NA | 4 | 4 | NA | NA |
|  |  | **13** | 04-May-15 | NA | 25 | 0.5 | 1 | NA | NA | 0.25 | 0.5 | NA | NA | 2 | 2 | NA | NA |
| **10** | **A070** | **642** | 09-Sep-13 | Absent | 5 | 0.25 | 0.25 | 4 | 4 | 0.125 | 0.25 | 8 | 16 | 2 | 2 | 0.5 | 1 |
|  |  | **285** | 14-Apr-14 | NA | 5 | 0.5 | 1 | NA | NA | 1 | 1 | NA | NA | 16 | 16 | NA | NA |
|  |  | **658** | 25-Jan-16 | NA | 5 | 0.5 | 2 | NA | NA | 0.5 | 0.5 | NA | NA | 4 | 32 | NA | NA |
| **11** | **A142** | **275** | 19-Jun-13 | A | 9 | 0.5 | 1 | 8 | 8 | 0.5 | 0.5 | 32 | 32 | 4 | 16 | 0.5 | 1 |
|  |  | **649** | 08-Sep-14 | NA | 9 | 0.5 | 2 | NA | NA | 0.25 | 0.5 | NA | NA | 4 | 16 | NA | NA |
|  |  | **519** | 02-Dec-15 | NA | 9 | 0.25 | 0.5 | NA | NA | 0.5 | 0.5 | NA | NA | 4 | 16 | NA | NA |
|  |  | **233** | 27-Oct-16 | NA | 9 | 0.5 | 1 | NA | NA | 0.5 | 0.5 | NA | NA | 4 | 8 | NA | NA |
| **12** | **A372** | **656** | 06-Oct-15 | Absent | 25 | 0.25 | 0.5 | 4 | 4 | 0.125 | 0.125 | 8 | 16 | 1 | 1 | 0.5 | 0.5 |
| **13** | **A283** | **516** | 30-Sep-13 | A | 8 | 0.5 | 0.5 | 4 | 4 | 0.25 | 0.25 | 8 | 8 | 4 | 4 | 0.5 | 1 |
|  |  | **652** | 05-Nov-14 | NA | 188 | 1 | 2 | NA | NA | 0.5 | 0.5 | NA | NA | 4 | ≥128 | NA | NA |
|  |  | **302** | 21-Jan-15 | NA | 8 | 1 | 1 | NA | NA | 1 | 1 | NA | NA | 4 | 8 | NA | NA |
|  |  | **226** | 21-Sep-16 | NA | 8 | 1 | 1 | NA | NA | 0.5 | 0.5 | NA | NA | 4 | ≥128 | NA | NA |
| **14** | **A284** | **280** | 06-Nov-13 | C | 121 | 1 | 1 | 8 | 8 | 0.5 | 0.5 | 32 | 32 | 8 | ≥128 | 1 | 2 |
|  |  | **648** | 06-Aug-14 | NA | 15 | 0.5 | 1 | NA | NA | 0.25 | 0.5 | NA | NA | 4 | ≥128 | NA | NA |
|  |  | **655** | 13-Jul-15 | NA | 15 | 0.5 | 1 | NA | NA | 0.5 | 0.5 | NA | NA | 4 | ≥128 | NA | NA |
|  |  | **19** | 14-Sep-16 | NA | Unknown | 0.5 | 1 | NA | NA | 0.5 | 0.5 | NA | NA | 4 | 16 | NA | NA |
| **15** | **A335** | **21** | 28-Jan-13 | C | 15 | 1 | 2 | 8 | 8 | 0.5 | 0.5 | 2 | 2 | 4 | ≥128 | 1 | 2 |
|  |  | **650** | 24-Sep-14 | NA | 15 | 1 | 2 | NA | NA | 0.5 | 1 | NA | NA | 8 | 64 | NA | NA |
|  |  | **402** | 26-Oct-15 | NA | 15 | 2 | 4 | NA | NA | 2 | 2 | NA | NA | 16 | ≥128 | NA | NA |
| **16** | **A017** | **643** | 08-Oct-13 | C | 30 | 0.5 | 2 | 4 | 4 | 0.25 | 0.25 | 8 | 8 | 2 | 8 | 0.5 | 1 |
| **17** | **A016** | **646** | 26-Feb-14 | C | 30 | 0.5 | 1 | 8 | 8 | 0.25 | 0.5 | 16 | 16 | 8 | 16 | 1 | 1 |
|  |  | **240**† | 01-Dec-16 | NA | 188 | 0.5 | 1 | NA | NA | 1 | 0.5 | NA | NA | 16 | 64 | NA | NA |
| **18** | **A044** | **647** | 17-Mar-14 | Absent | 1 | 0.25 | 0.5 | 4 | 4 | 0.125 | 0.25 | 16 | 32 | 4 | 4 | 0.25 | 1 |
|  |  | **29** | 02-Sep-15 | NA | 1 | 0.5 | 0.5 | NA | NA | 0.5 | 0.5 | NA | NA | 4 | 16 | NA | NA |
| **19** | **A366** | **273** | 03-Jun-13 | A | 15 | 0.25 | 0.5 | 4 | 8 | 0.5 | 0.5 | 32 | 32 | 2 | 2 | 1 | 1 |
|  |  | **296** | 22-Oct-14 | NA | 15 | 1 | 2 | NA | NA | 1 | 1 | NA | NA | 4 | 8 | NA | NA |
|  |  | **236** | 18-Nov-16 | NA | 1 | 0.5 | 0.5 | NA | NA | 0.5 | 0.5 | NA | NA | 8 | 16 | NA | NA |
| **20** | **A272** | **34** | 13-Nov-13 | D | 15 | 0.5 | 1 | 4 | 4 | 0.25 | 0.25 | 1 | 2 | 1 | 1 | 0.25 | 1 |
| **21** | **A361** | **702** | 22-Jul-13 | A | Unknown | 0.5 | 2 | 4 | 4 | 0.25 | 0.25 | 8 | 8 | 2 | 4 | 0.5 | 1 |
|  |  | **708** | 05-Jan-15 | NA | 30 | 0.5 | 2 | NA | NA | 0.25 | 0.5 | NA | NA | 2 | 4 | NA | NA |
|  |  | **717** | 03-Oct-16 | NA | 30 | 1 | 4 | NA | NA | 0.5 | 1 | NA | NA | 2 | 4 | NA | NA |
| **22** | **A384** | **38** | 06-Aug-15 | B | 8 | 0.5 | 1 | 4 | 8 | 0.125 | 0.25 | 1 | 2 | 2 | ≥128 | 4 | 8 |
| **23** | **A065** | **706**† | 29-Sep-14 | C | 30 | 0.25 | 0.25 | 4 | 4 | 0.25 | 0.25 | 16 | 32 | 4 | 2 | 0.5 | 1 |
| **24** | **A126** | **701** | 09-Apr-13 | D | Unknown | 0.5 | 1 | 4 | 8 | 0.25 | 0.25 | 16 | 16 | 2 | 2 | 1 | 1 |
| **25** | **A368** | **699** | 01-Jan-13 | C | 9 | 0.5 | 1 | 8 | 8 | 0.25 | 0.25 | 8 | 8 | 2 | 4 | 0.5 | 1 |
| **26** | **A041** | **704** | 09-Dec-13 | Absent | 5 | 1 | 4 | 4 | 4 | 0.125 | 0.125 | 8 | 8 | 1 | 1 | 0.25 | 0.5 |
|  |  | **720** | 12-Nov-14 | NA | 5 | 0.5 | 0.5 | NA | NA | 0.5 | 0.5 | NA | NA | 0.5 | 0.5 | NA | NA |
|  |  | **714** | 09-Nov-15 | NA | 5 | 0.5 | 0.5 | NA | NA | 0.25 | 0.5 | NA | NA | 2 | 4 | NA | NA |
|  |  | **235** | 16-Nov-16 | NA | 5 | 0.25 | 0.5 | NA | NA | 0.5 | 1 | NA | NA | 2 | 2 | NA | NA |
| **27** | **A386** | **46**† | 03-Sep-14 | B | 121 | 0.5 | 1 | 4 | 4 | 0.25 | 0.125 | 1 | 1 | 2 | 4 | 0.5 | 1 |
| **28** | **A387** | **710** | 20-May-15 | C | 15 | 0.5 | 1 | 4 | 8 | 0.25 | 0.25 | 32 | 32 | 2 | 32 | 0.5 | 2 |
|  |  | **48** | 13-Jan-16 | NA | 45 | 0.5 | 1 | NA | NA | 0.5 | 0.5 | NA | NA | 2 | 4 | NA | NA |
| **29** | **A090** | **700** | 11-Feb-13 | C | 1 | 0.5 | 1 | 4 | 8 | 0.125 | 0.125 | 8 | 16 | 4 | 16 | 0.5 | 1 |
|  |  | **292** | 25-Aug-14 | NA | Unknown | 0.5 | 1 | NA | NA | 1 | 1 | NA | NA | 8 | 64 | NA | NA |
|  |  | **711** | 02-Sep-15 | NA | 1 | 0.5 | 1 | NA | NA | 0.25 | 0.5 | NA | NA | 4 | 32 | NA | NA |
|  |  | **238** | 23-Nov-16 | NA | 1 | 0.5 | 1 | NA | NA | 0.5 | 0.5 | NA | NA | 16 | 32 | NA | NA |
| **30** | **A325** | **715** | 28-Mar-16 | C | 25 | 0.5 | 1 | 4 | 8 | 0.25 | 0.25 | 32 | 32 | 4 | 16 | 1 | 1 |
| **31** | **A020** | **279** | 09-Sep-13 | C | 45 | 0.5 | 1 | 8 | 8 | 0.5 | 0.5 | 32 | 32 | 4 | 8 | 1 | 1 |
|  |  | **522** | 22-Aug-16 | NA | 8 | 0.5 | 0.5 | NA | NA | 0.25 | 0.5 | NA | NA | 2 | 8 | NA | NA |
| **32** | **A042** | **795** | 07-Jan-13 | C | 5 | 0.5 | 1 | 8 | 8 | 0.125 | 0.125 | 8 | 8 | 4 | 4 | 1 | 2 |
| **33** | **A043** | **703** | 18-Nov-13 | A | 5 | 1 | 4 | 1 | 2 | 0.5 | 0.5 | 8 | 8 | 4 | 16 | 0.25 | 0.5 |
|  |  | **707** | 01-Dec-14 | NA | 8 | 1 | 1 | NA | NA | 0.5 | 0.5 | NA | NA | 4 | 8 | NA | NA |
| **34** | **A290** | **799** | 18-Sep-13 | A | 5 | 2 | 8 | 2 | 2 | 0.25 | 0.25 | 16 | 16 | 2 | 4 | 1 | 2 |
|  |  | **809** | 15-Oct-14 | NA | 5 | 2 | 16 | NA | NA | 0.5 | 0.5 | NA | NA | 4 | 16 | NA | NA |
|  |  | **814** | 03-Nov-15 | NA | 5 | 1 | ≥64 | NA | NA | 0.5 | 1 | NA | NA | 2 | 64 | NA | NA |
| **35** | **A388** | **712** | 16-Sep-15 | C | 398 | 0.5 | 1 | 4 | 8 | 0.5 | 0.5 | 32 | 32 | 2 | 8 | 0.5 | 1 |
|  |  | **718** | 01-Dec-16 | NA | 398 | 0.5 | 1 | NA | NA | 0.5 | 1 | NA | NA | 4 | 16 | NA | NA |
| **36** | **A389** | **713** | 30-Sep-15 | C | 45 | 0.5 | 1 | 4 | 8 | 0.25 | 0.25 | 16 | 32 | 2 | 8 | 0.5 | 1 |
|  |  | **716** | 15-Aug-16 | NA | Unknown | 0.5 | 0.5 | NA | NA | 0.25 | 0.5 | NA | NA | 2 | 2 | NA | NA |
| **37** | **A390** | **62**† | 10-Sep-14 | D | 15 | 0.25 | 0.5 | 4 | 8 | 0.125 | 0.25 | 0.5 | 4 | 2 | 1 | 0.25 | 1 |
| **38** | **A369** | **818** | 03-Oct-16 | A | Unknown | 2 | 8 | 8 | 16 | 0.5 | 0.5 | 32 | 32 | 4 | 4 | 1 | 4 |
| **39** | **A391** | **804** | 05-Feb-14 | A | 15 | 1 | 2 | NA | NA | 0.25 | 0.5 | NA | NA | 2 | 16 | NA | NA |
|  |  | **304** | 04-Mar-15 | NA | 15 | 1 | 4 | NA | NA | 1 | 1 | NA | NA | 8 | 64 | NA | NA |
| **40** | **A291** | **67** | 17-Jul-13 | C | 30 | 0.25 | 0.5 | 4 | 8 | 0.125 | 0.125 | 1 | 2 | 2 | 2 | 0.25 | 1 |
|  |  | **68** | 17-Nov-14 | NA | 30 | 1 | 2 | NA | NA | 0.5 | 1 | NA | NA | 4 | 4 | NA | NA |
| **41** | **A367** | **802**† | 25-Nov-13 | C | Unknown | 2 | 1 | 4 | 4 | 0.25 | 0.25 | 32 | 32 | 0.5 | 2 | 0.5 | 1 |
|  |  | **286** | 05-May-14 | NA | 5 | 1 | 2 | NA | NA | 1 | 1 | NA | NA | 16 | ≥128 | NA | NA |
| **43** | **A293** | **270**† | 14-Jan-13 | Absent | 15 | 2 | 1 | 8 | 8 | 1 | 0.5 | 32 | 32 | 4 | 4 | 1 | 1 |
|  |  | **808** | 27-Aug-14 | NA | 15 | 1 | 4 | NA | NA | 1 | 1 | NA | NA | 16 | ≥128 | NA | NA |
|  |  | **223** | 24-Aug-16 | NA | 30 | 0.5 | 1 | NA | NA | 0.5 | 0.5 | NA | NA | 4 | 4 | NA | NA |
| **44** | **A046** | **796** | 23-Jan-13 | A | 5 | 0.5 | 1 | 8 | 8 | 0.125 | 0.25 | 16 | 16 | 2 | 2 | 0.5 | 1 |
|  |  | **805**† | 14-Feb-14 | NA | 5 | 1 | 0.5 | NA | NA | 0.25 | 0.5 | NA | NA | 2 | 4 | NA | NA |
|  |  | **810** | 30-Mar-15 | NA | 5 | 0.5 | 0.5 | NA | NA | 0.5 | 0.5 | NA | NA | 8 | 32 | NA | NA |
|  |  | **222** | 22-Aug-16 | NA | 5 | 0.5 | 0.5 | NA | NA | 0.25 | 0.5 | NA | NA | 4 | 16 | NA | NA |
| **45** | **A360** | **797** | 01-May-13 | C | Unknown | 0.5 | 1 | 4 | 4 | 0.125 | 0.125 | 16 | 16 | 4 | 4 | 1 | 1 |
|  |  | **297** | 17-Nov-14 | NA | Unknown | 0.5 | 16 | NA | NA | 0.5 | 1 | NA | NA | 4 | 4 | NA | NA |
|  |  | **816** | 09-Nov-15 | NA | Unknown | 1 | 8 | NA | NA | 0.5 | 0.5 | NA | NA | 4 | 8 | NA | NA |
|  |  | **227** | 29-Sep-16 | NA | 9 | 0.25 | 0.5 | NA | NA | 0.125 | 0.25 | NA | NA | 2 | 2 | NA | NA |
| **46** | **A294** | **287**† | 02-Jun-14 | C | 5 | 0.5 | 1 | 4 | 8 | 0.5 | 0.25 | 16 | 32 | 4 | 32 | 0.5 | 1 |
|  |  | **812** | 10-Aug-15 | NA | 5 | 0.5 | 1 | NA | NA | 0.25 | 0.5 | NA | NA | 8 | 64 | NA | NA |
|  |  | **78** | 24-Aug-16 | NA | 5 | 0.5 | 2 | NA | NA | 0.5 | 0.5 | NA | NA | 4 | >128 | NA | NA |
| **47** | **A295** | **798** | 08-May-13 | A | 5 | 0.5 | 0.5 | 8 | 8 | 0.25 | 0.25 | 32 | 32 | 1 | 2 | 1 | 2 |
| **48** | **A394** | **801** | 06-Nov-13 | C | 15 | 0.5 | 0.5 | 4 | 4 | 0.25 | 0.5 | 32 | 32 | 4 | ≥128 | 0.5 | 0.5 |
|  |  | **80** | 03-Nov-16 | NA | 15 | 1 | 2 | NA | NA | 0.5 | 0.5 | NA | NA | 8 | 32 | NA | NA |
| **49** | **A341** | **84** | 24-Nov-14 | Absent | 1 | 0.125 | 0.125 | 4 | 4 | 0.125 | 0.25 | 1 | 1 | 1 | 1 | 0.5 | 1 |
|  |  | **85**† | 07-Jan-15 | NA | 1 | 0.5 | 0.5 | NA | NA | 0.5 | 0.5 | NA | NA | 4 | 2 | NA | NA |
| **50** | **A059** | **87** | 05-Apr-13 | B | 25 | 0.5 | 1 | 8 | 8 | 0.25 | 0.25 | 1 | 2 | 4 | 8 | 2 | 2 |
|  |  | **806** | 31-Mar-14 | NA | Unknown | 1 | 2 | NA | NA | 0.5 | 0.5 | NA | NA | 8 | 16 | NA | NA |
|  |  | **89** | 07-Jan-15 | NA | 8 | 1 | 1 | NA | NA | 0.5 | 0.5 | NA | NA | 8 | 8 | NA | NA |
|  |  | **224** | 12-Sep-16 | NA | 8 | 1 | 2 | NA | NA | 0.5 | 1 | NA | NA | 4 | ≥128 | NA | NA |
| **51** | **A395** | **815**† | 03-Nov-15 | A | 5 | 0.5 | 0.25 | 8 | 8 | 0.25 | 0.25 | 32 | 32 | 1 | 1 | 1 | 2 |
|  |  | **820** | 19-Oct-16 | NA | 5 | 0.25 | 0.25 | NA | NA | 0.25 | 0.5 | NA | NA | 2 | 2 | NA | NA |
| **52** | **A298** | **800** | 09-Oct-13 | C | Unknown | 0.5 | 1 | 2 | 8 | 0.25 | 0.25 | 16 | 16 | 0.5 | 2 | 0.125 | 0.5 |
|  |  | **283** | 24-Feb-14 | NA | Unknown | 0.5 | 0.5 | NA | NA | 0.5 | 0.5 | NA | NA | 8 | 8 | NA | NA |
| **53** | **A057** | **918** | 25-Mar-13 | Absent | 1 | 0.5 | 8 | 4 | 4 | 0.125 | 0.25 | 16 | 32 | 1 | 1 | 0.5 | 1 |
|  |  | **300** | 03-Dec-14 | NA | 1 | 0.25 | 0.5 | NA | NA | 1 | 1 | NA | NA | 2 | 2 | NA | NA |
| **54** | **A143** | **919** | 24-Apr-14 | A | 30 | 0.5 | 2 | 8 | 8 | 0.25 | 0.25 | 32 | 32 | 1 | 1 | 0.5 | 1 |
| **55** | **A344** | **939** | 12-Sep-16 | Absent | 5 | 1 | 8 | 4 | 8 | 0.25 | 0.25 | 32 | 32 | 2 | 4 | 1 | 1 |
| **56** | **A300** | **517** | 15-Jan-14 | C | 15 | 1 | 2 | 4 | 4 | 0.25 | 0.5 | 16 | 16 | 16 | ≥128 | 2 | 2 |
|  |  | **932** | 27-Jul-15 | NA | 15 | 1 | 4 | NA | NA | 0.5 | 0.5 | NA | NA | 16 | ≥128 | NA | NA |
|  |  | **1158** | 27-Jul-16 | NA | Unknown | 1 | 4 | NA | NA | 1 | 1 | NA | NA | 8 | ≥128 | NA | NA |
| **57** | **A219** | **276** | 15-Jul-13 | C | 45 | 0.5 | 1 | 4 | 8 | 0.25 | 0.25 | 32 | 32 | 4 | ≥128 | 1 | 2 |
|  |  | **926** | 27-Aug-14 | NA | 45 | 1 | 2 | NA | NA | 0.5 | 1 | NA | NA | 8 | ≥128 | NA | NA |
|  |  | **100** | 06-Jan-16 | NA | 45 | 0.25 | 0.25 | NA | NA | 0.25 | 0.5 | NA | NA | 2 | 4 | NA | NA |
| **58** | **A301** | **277** | 07-Aug-13 | C | 45 | 0.5 | 1 | 4 | 4 | 0.25 | 0.5 | 16 | 32 | 4 | ≥128 | 0.5 | 1 |
|  |  | **301** | 22-Dec-14 | NA | 45 | 0.5 | 0.5 | NA | NA | 0.5 | 1 | NA | NA | 2 | 4 | NA | NA |
| **59** | **A340** | **923** | 12-May-14 | A | 30 | 2 | 2 | 4 | 4 | 0.25 | 0.25 | 16 | 32 | 2 | 4 | 0.5 | 1 |
|  |  | **103** | 09-Sep-15 | NA | 30 | 0.5 | 2 | NA | NA | 0.25 | 0.5 | NA | NA | 4 | 4 | NA | NA |
| **60** | **A086** | **920** | 15-May-13 | A | 30 | 0.5 | 2 | 4 | 4 | 0.25 | 0.5 | 16 | 32 | 2 | 2 | 0.5 | 1 |
|  |  | **924** | 23-Jun-14 | NA | 30 | 0.5 | ≥64 | NA | NA | 0.5 | 0.5 | NA | NA | 4 | 16 | NA | NA |
|  |  | **936** | 01-Feb-16 | NA | 30 | 1 | 8 | NA | NA | 0.5 | 0.5 | NA | NA | 4 | 16 | NA | NA |
| **62** | **A345** | **108** | 05-May-14 | C | 5 | 0.5 | 0.5 | 4 | 8 | 0.125 | 0.125 | 0.5 | 4 | 1 | 1 | 0.5 | 2 |
| **63** | **A127** | **274** | 12-Jun-13 | Absent | 5 | 0.5 | 0.5 | 4 | 4 | 1 | 1 | 32 | 32 | 2 | 4 | 0.5 | 1 |
|  |  | **518** | 22-Jan-14 | NA | 5 | 0.5 | 0.5 | NA | NA | 0.5 | 0.5 | NA | NA | 2 | 2 | NA | NA |
|  |  | **930**† | 23-Feb-15 | NA | 5 | 0.5 | 0.5 | NA | NA | 0.5 | 1 | NA | NA | 4 | 2 | NA | NA |
|  |  | **940** | 24-Oct-16 | NA | 5 | 0.5 | 0.5 | NA | NA | 0.5 | 1 | NA | NA | 4 | 4 | NA | NA |
| **64** | **A397** | **114** | 24-Aug-15 | B | 5 | 0.5 | 2 | 4 | 4 | 0.125 | 0.125 | 16 | 32 | 2 | 2 | 0.5 | 1 |
| **65** | **A026** | **925** | 25-Jun-14 | Absent | 45 | 0.25 | 0.5 | 4 | 4 | 0.25 | 0.25 | 32 | 32 | 2 | 2 | 0.5 | 0.5 |
| **66** | **A399** | **284** | 10-Mar-14 | Absent | 121 | 0.5 | 0.5 | 4 | 8 | 0.125 | 0.25 | 0.5 | 2 | 4 | 8 | 0.5 | 1 |
|  |  | **305** | 01-May-15 | NA | 121 | 1 | 1 | NA | NA | 0.5 | 1 | NA | NA | 4 | 8 | NA | NA |
|  |  | **221** | 15-Aug-16 | NA | 121 | 0.5 | 0.5 | NA | NA | 0.5 | 0.5 | NA | NA | 4 | 8 | NA | NA |
| **67** | **A373** | **118**† | 10-Feb-14 | C | 5 | 0.5 | 0.5 | 4 | 8 | 0.25 | 0.125 | 1 | 2 | 1 | 1 | 0.25 | 2 |
|  |  | **303** | 18-Feb-15 | NA | 5 | 8 | ≥64 | NA | NA | 4 | 8 | NA | NA | 16 | 32 | NA | NA |
| **68** | **A013** | **927** | 01-Oct-14 | B | 30 | 0.5 | 1 | 4 | 8 | 0.125 | 0.25 | 16 | 32 | 2 | 8 | 0.5 | 1 |
|  |  | **311** | 20-Apr-16 | NA | 30 | 1 | 2 | NA | NA | 1 | 1 | NA | NA | 4 | 16 | NA | NA |
| **69** | **A371** | **124** | 21-Jan-13 | D | 45 | 0.25 | 0.5 | 4 | 4 | 0.125 | 0.25 | 1 | 1 | 2 | 4 | 1 | 1 |
|  |  | **406** | 17-Sep-14 | NA | Unknown | 0.5 | 1 | NA | NA | 1 | 1 | NA | NA | 4 | 16 | NA | NA |
|  |  | **935** | 21-Oct-15 | NA | 45 | 0.5 | 0.5 | NA | NA | 0.5 | 0.5 | NA | NA | 4 | 16 | NA | NA |
| **70** | **A072** | **125** | 14-Aug-13 | C | 8 | 0.5 | 1 | 8 | 16 | 0.25 | 0.5 | 1 | 2 | 2 | 32 | 16 | 32 |
|  |  | **288** | 05-Jun-14 | NA | 8 | 2 | 2 | NA | NA | 1 | 1 | NA | NA | 16 | ≥128 | NA | NA |
|  |  | **933**† | 29-Jul-15 | NA | 8 | 0.5 | 1 | NA | NA | 0.5 | 0.25 | NA | NA | 8 | 32 | NA | NA |
|  |  | **127** | 20-May-16 | NA | 8 | 0.5 | 1 | NA | NA | 0.5 | 0.5 | NA | NA | 4 | 8 | NA | NA |
| **71** | **A144** | **272** | 13-Feb-13 | C | 5 | 0.5 | 0.5 | 4 | 4 | 0.25 | 0.25 | 16 | 16 | 4 | 32 | 0.5 | 1 |
|  |  | **922** | 23-Apr-14 | NA | 5 | 0.5 | 1 | NA | NA | 0.5 | 0.5 | NA | NA | 8 | 32 | NA | NA |
|  |  | **310** | 21-Mar-16 | NA | 15 | 0.5 | 1 | NA | NA | 1 | 1 | NA | NA | 2 | 16 | NA | NA |
| **72** | **A329** | **129** | 29-Oct-14 | C | 15 | 0.5 | 2 | 8 | 8 | 0.25 | 0.25 | 2 | 4 | 2 | 32 | 1 | 2 |
|  |  | **934** | 31-Aug-15 | NA | 15 | 1 | 2 | NA | NA | 0.5 | 0.5 | NA | NA | 8 | ≥128 | NA | NA |
|  |  | **942**† | 01-Dec-16 | NA | 15 | 1 | 2 | NA | NA | 1 | 0.5 | NA | NA | 8 | 64 | NA | NA |
| **73** | **A374** | **135**† | 08-Jul-15 | A | 45 | 0.25 | 0.5 | 4 | 4 | 0.25 | 0.125 | 1 | 1 | 2 | 0.5 | 0.5 | 2 |
| **74** | **A312** | **1093** | 06-May-13 | Absent | 25 | 0.5 | 0.5 | 4 | 4 | 0.25 | 0.25 | 32 | 64 | 8 | 16 | 0.5 | 1 |
|  |  | **1116** | 04-Jun-14 | NA | 25 | 0.5 | 0.5 | NA | NA | 0.5 | 1 | NA | NA | 8 | 4 | NA | NA |
| **75** | **A275** | **143** | 11-Feb-13 | D | 45 | 0.5 | 1 | 4 | 8 | 0.25 | 0.25 | 1 | 2 | 2 | 4 | 0.5 | 1 |
|  |  | **144** | 26-Aug-15 | NA | 8 | 0.5 | 1 | NA | NA | 0.5 | 0.5 | NA | NA | 4 | 16 | NA | NA |
| **76** | **A375** | **1154** | 26-May-16 | Absent | 15 | 0.5 | 1 | 4 | 8 | 0.25 | 0.25 | 32 | 32 | 4 | 4 | 1 | 2 |
| **77** | **A337** | **1097** | 10-Jul-13 | Absent | 106 | 0.25 | 0.25 | 4 | 4 | 0.125 | 0.125 | 16 | 16 | 4 | 4 | 0.5 | 1 |
|  |  | **299** | 25-Nov-14 | NA | 15 | 0.5 | 0.5 | NA | NA | 0.5 | 0.5 | NA | NA | 4 | 4 | NA | NA |
| **78** | **A063** | **513** | 13-Mar-13 | A | 30 | 0.5 | 1 | 4 | 8 | 0.25 | 0.25 | 16 | 32 | 4 | 4 | 0.5 | 1 |
|  |  | **1114** | 21-May-14 | NA | 30 | 1 | 2 | NA | NA | 0.5 | 0.5 | NA | NA | 8 | 8 | NA | NA |
|  |  | **1135** | 01-Jun-15 | NA | 30 | 1 | 4 | NA | NA | 0.25 | 0.5 | NA | NA | 4 | 8 | NA | NA |
|  |  | **313** | 16-May-16 | NA | 30 | 1 | 8 | NA | NA | 1 | 1 | NA | NA | 4 | 8 | NA | NA |
| **79** | **A402** | **147** | 28-Sep-15 | B | Unknown | 1 | 2 | 2 | 4 | 0.5 | 0.5 | 16 | 32 | 2 | 4 | 0.5 | 1 |
|  |  | **149** | 16-Nov-16 | NA | 25 | 0.5 | 0.5 | NA | NA | 0.25 | 0.5 | NA | NA | 2 | 2 | NA | NA |
| **80** | **A064** | **1088** | 06-Mar-13 | A | Unknown | 0.25 | 1 | 2 | 2 | 0.125 | 0.25 | 4 | 16 | 2 | ≥128 | 0.5 | 4 |
|  |  | **1108** | 17-Mar-14 | NA | 25 | 1 | ≥64 | NA | NA | 0.5 | 1 | NA | NA | 8 | ≥128 | NA | NA |
| **81** | **A011** | **1102** | 30-Oct-13 | C | 15 | 1 | 8 | 8 | 8 | 0.125 | 0.25 | 32 | 32 | 16 | ≥128 | 2 | 2 |
|  |  | **1121** | 22-Oct-14 | NA | 5 | 0.5 | 0.5 | NA | NA | 0.5 | 1 | NA | NA | 4 | 4 | NA | NA |
|  |  | **1150**† | 11-Jan-16 | NA | 5 | 8 | 1 | NA | NA | 0.5 | 0.5 | NA | NA | 4 | ≥128 | NA | NA |
| **82** | **A039** | **1098** | 31-Jul-13 | A | 30 | 1 | 4 | 4 | 8 | 0.25 | 0.25 | 16 | 16 | 8 | 16 | 1 | 1 |
|  |  | **1109** | 31-Mar-14 | NA | 30 | 1 | 2 | NA | NA | 1 | 1 | NA | NA | 8 | 8 | NA | NA |
|  |  | **230** | 17-Oct-16 | NA | 106 | 0.5 | 1 | NA | NA | 0.5 | 0.5 | NA | NA | 2 | 4 | NA | NA |
| **83** | **A071** | **1085** | 13-Feb-13 | Absent | 1 | 0.5 | 8 | 2 | 2 | 0.125 | 0.25 | 0.25 | 0.5 | 16 | 64 | 1 | 1 |
|  |  | **1106** | 10-Feb-14 | NA | 1 | 0.5 | 0.5 | NA | NA | 0.5 | 0.5 | NA | NA | 4 | ≥128 | NA | NA |
|  |  | **1131** | 08-Apr-15 | NA | 1 | 0.125 | 0.25 | NA | NA | 0.5 | 0.5 | NA | NA | 1 | 1 | NA | NA |
|  |  | **1159** | 10-Aug-16 | NA | 1 | 0.5 | 1 | NA | NA | 0.5 | 1 | NA | NA | 16 | ≥128 | NA | NA |
| **84** | **A403** | **1128** | 09-Feb-15 | Absent | 15 | 1 | 4 | 8 | 8 | 0.5 | 0.5 | 2 | 4 | 4 | 4 | 1 | 2 |
| **85** | **A364** | **164** | 05-Aug-15 | B | 1 | 1 | 2 | 8 | 8 | 1 | 1 | 4 | 4 | 8 | ≥128 | 2 | 2 |
|  |  | **1160** | 08-Sep-16 | NA | 1 | 8 | 8 | NA | NA | 1 | 1 | NA | NA | 16 | ≥128 | NA | NA |
| **86** | **A319** | **1117**† | 07-Jul-14 | Absent | 5 | 2 | 8 | 8 | 8 | 1 | 0.5 | 16 | 32 | 8 | 16 | 0.5 | 1 |
|  |  | **1142**† | 19-Oct-15 | NA | 5 | 1 | 0.5 | NA | NA | 1 | 0.5 | NA | NA | 8 | 4 | NA | NA |
|  |  | **1164** | 05-Oct-16 | NA | 5 | 0.5 | 0.5 | NA | NA | 0.5 | 0.5 | NA | NA | 4 | 8 | NA | NA |
| **87** | **A320** | **167** | 03-Dec-14 | D | 5 | 1 | 4 | 4 | 8 | 0.5 | 0.5 | 16 | 32 | 2 | 4 | 1 | 1 |
|  |  | **1140** | 29-Jun-15 | NA | 5 | 1 | 1 | NA | NA | 0.5 | 0.5 | NA | NA | 4 | 16 | NA | NA |
| **88** | **A404** | **1149** | 21-Dec-15 | A | Unknown | 0.5 | 0.5 | 8 | 8 | 0.5 | 0.5 | 16 | 32 | 8 | 32 | 0.5 | 1 |
| **89** | **A405** | **159** | 31-Aug-15 | C | 15 | 0.25 | 0.5 | 8 | 8 | 0.25 | 0.25 | 16 | 32 | 2 | 2 | 2 | 2 |
| **90** | **A376** | **153** | 04-Feb-14 | D | 5 | 0.5 | 2 | 8 | 8 | 0.25 | 0.25 | 1 | 4 | 1 | 1 | 0.5 | 2 |
|  |  | **154** | 24-Jun-15 | NA | 15 | 0.5 | 0.5 | NA | NA | 0.25 | 0.5 | NA | NA | 4 | ≥128 | NA | NA |
|  |  | **171** | 17-Aug-16 | NA | 5 | 0.5 | 0.5 | NA | NA | 0.5 | 0.5 | NA | NA | 4 | ≥128 | NA | NA |
| **91** | **A323** | **1100** | 27-Sep-13 | Absent | 25 | 0.5 | 0.5 | 4 | 8 | 0.25 | 0.25 | 16 | 32 | 8 | 8 | 0.5 | 0.5 |
|  |  | **1119** | 06-Oct-14 | NA | 25 | 0.5 | 1 | NA | NA | 0.5 | 1 | NA | NA | 4 | 4 | NA | NA |
|  |  | **1145** | 02-Nov-15 | NA | 25 | 1 | 32 | NA | NA | 0.5 | 1 | NA | NA | 4 | ≥128 | NA | NA |
|  |  | **237** | 21-Nov-16 | NA | 25 | 0.5 | 0.5 | NA | NA | 0.5 | 1 | NA | NA | 8 | 16 | NA | NA |
| **92** | **A370** | **1103** | 02-Dec-13 | Absent | Unknown | 2 | 8 | 4 | 4 | 0.25 | 0.25 | 0.5 | 0.5 | 0.25 | 0.5 | 0.25 | 0.25 |
|  |  | **1122** | 05-Nov-14 | NA | Unknown | 0.5 | 0.5 | NA | NA | 0.25 | 0.5 | NA | NA | 4 | 4 | NA | NA |
|  |  | **1143** | 28-Oct-15 | NA | Unknown | 0.5 | 4 | NA | NA | 0.5 | 0.5 | NA | NA | 4 | 4 | NA | NA |
|  |  | **241** | 01-Dec-16 | NA | Unknown | 0.5 | 0.5 | NA | NA | 0.5 | 1 | NA | NA | 4 | 4 | NA | NA |
| **93** | **A276** | **1104** | 11-Dec-13 | C | 5 | 1 | 1 | 4 | 4 | 0.25 | 0.5 | 16 | 16 | 8 | ≥128 | 1 | 1 |
|  |  | **291** | 23-Jul-14 | NA | 5 | 0.5 | 0.5 | NA | NA | 1 | 1 | NA | NA | 8 | ≥128 | NA | NA |
|  |  | **1134** | 27-May-15 | NA | 5 | 0.5 | 1 | NA | NA | 0.25 | 0.5 | NA | NA | 4 | ≥128 | NA | NA |
|  |  | **1156** | 11-Jul-16 | NA | 188 | 0.5 | 1 | NA | NA | 0.5 | 0.5 | NA | NA | 4 | ≥128 | NA | NA |
| **94** | **A324** | **1105** | 29-Jan-14 | C | 97 | 0.5 | 4 | 8 | 8 | 1 | 1 | 16 | 16 | 16 | 32 | 1 | 1 |
|  |  | **1133** | 13-May-15 | NA | 97 | 2 | 16 | NA | NA | 1 | 1 | NA | NA | 4 | 32 | NA | NA |
|  |  | **314** | 13-Jul-16 | NA | 97 | 2 | 4 | NA | NA | 2 | 2 | NA | NA | 8 | 64 | NA | NA |
| **95** | **A328** | **177**† | 28-Aug-13 | A | 45 | 0.5 | 0.5 | 4 | 4 | 0.5 | 1 | 16 | 32 | 8 | 4 | 0.5 | 1 |
| **96** | **A004** | **1090** | 31-Mar-13 | A | 398 | 1 | 4 | 4 | 4 | 0.25 | 0.25 | 16 | 16 | 8 | 16 | 0.5 | 2 |
|  |  | **1112** | 14-May-14 | NA | 398 | 1 | 1 | NA | NA | 0.5 | 1 | NA | NA | 8 | 32 | NA | NA |
|  |  | **1167**† | 09-Nov-16 | NA | 398 | 0.5 | 1 | NA | NA | 1 | 0.5 | NA | NA | 8 | 64 | NA | NA |
| **97** | **A003** | **1123** | 05-Nov-14 | A | 30 | 1 | 1 | 8 | 8 | 0.5 | 0.5 | 32 | 64 | 8 | 32 | 1 | 2 |
|  |  | **307** | 14-Sep-15 | NA | Unknown | 8 | ≥64 | NA | NA | 4 | 8 | NA | NA | 16 | ≥128 | NA | NA |
| **98** | **A377** | **1096** | 03-Jul-13 | A | 30 | 0.5 | 4 | 4 | 4 | 0.125 | 0.25 | 16 | 32 | 8 | 16 | 0.5 | 1 |
|  |  | **181** | 20-Aug-14 | NA | 30 | 0.5 | 1 | NA | NA | 0.5 | 0.5 | NA | NA | 4 | 4 | NA | NA |
|  |  | **182** | 26-Apr-16 | NA | 30 | 0.5 | 1 | NA | NA | 0.5 | 0.5 | NA | NA | 4 | 64 | NA | NA |

*Patients #42, #61, and #99 were excluded from analysis, as described in the main “Results”.

**Antibiotic Abbreviations: CZ: cefazolin, FEP: cefepime, MEM: meropenem, CAZ: ceftazidime, TZP: piperacillin-tazobactam, CLO: cloxacillin.

† Isolates showed noticeable difficulty resuspending in saline.

**Table E8: Baseline clinical characteristics of 96 persons with CF as potential risk factors for having at least one IE-positive MSSA strain for cefazolin and piperacillin-tazobactam throughout the study duration.** Values are reported as percentages of the total number of patients (n=96), where relevant quantities for each characteristic are noted in the leftmost column.

| **Patient Characteristics** | **Cefazolin** | | | **Piperacillin-Tazobactam** | | |
| --- | --- | --- | --- | --- | --- | --- |
|  | **IE +**  **MSSA** | **IE -**  **MSSA** | ***P*** | **IE +**  **MSSA** | **IE –**  **MSSA** | ***P*** |
| Age (median (IQR), years) | 26.3  (20.4-32.2) | 25.1  (21.3-35.5) | 0.1594 | 25.5  (21.7-31.0) | 25.1  (19.6-41.4) | 0.9752 |
| Female (%, n=47) | 38.3 (n=18) | 61.7 (n=29) | 0.5208 | 51.1 (n=24) | 48.9 (n=23) | 0.4127 |
| Male (%, n=49) | 36.7 (n=18) | 63.3 (n=31) | 0.5208 | 40.8 (n=20) | 59.2 (n=29) | 0.2110 |
| Pancreatic Insufficient  (%, n=74) | 40.5 (n=30) | 59.5 (n=44) | 0.1910 | 44.6 (n=33) | 55.4 (n=41) | 0.4180 |
| Baseline FEV1  (median (IQR), % predicted) | 72.5  (58.0-89.5) | 68.0  (46.5-82.5) | 0.1135 | 72.5  (53.5-86.5) | 67.0  (46.5-85.5) | 0.3125 |
| Baseline BMI  (median (IQR), kg/m^2^) | 21.7  (19.7-24.7) | 20.9  (19.5-23.4) | 0.3460 | 21.5  (19.7-23.6) | 21.4  (19.6-24.7) | 0.8808 |
| Chronic Medications:  -Oral Antibiotics (n=12)  -Inhaled Antibiotics (n=38) | 33.3 (n=4)  26.3 (n=10) | 66.7 (n=8)  47.4 (n=18) | 0.5083  0.5033 | 41.7 (n=5)  50.0 (n=19) | 58.3 (n=7)  44.7 (n=17) | 0.5027  0.1987 |

**Figure E1: Pulsed-field gel electrophoresis dendrogram of 223 CF-derived MSSA isolates.** Isolates are identified by A (ID, Table 7) number, SA (isolate) number, and collection date (dd/mm/yyyy). Identified pulsotypes are indicated in the rightmost column, where blanks indicate unknown pulsotype.

**Literature Cited:**

1. Clinical and Laboratory Standards Institute (CLSI). *M100-S19: Performance Standards for Antimicrobial Susceptibility Testing*. Clinical and Laboratory Standards Institute; 2009.

2. Mossman AK, Svishchuk J, Waddell BJM, et al. Staphylococcus aureus in Non-Cystic Fibrosis Bronchiectasis: Prevalence and Genomic Basis of High Inoculum Beta-Lactam Resistance. *Ann Am Thorac Soc*. Published online February 25, 2022. doi:10.1513/AnnalsATS.202108-965OC

3. Wang SK, Gilchrist A, Loukitcheva A, et al. Prevalence of a Cefazolin Inoculum Effect Associated with blaZ Gene Types among Methicillin-Susceptible Staphylococcus aureus Isolates from Four Major Medical Centers in Chicago. *Antimicrob Agents Chemother*. 2018;62(8). doi:10.1128/AAC.00382-18

4. Voladri RK, Kernodle DS. Characterization of a chromosomal gene encoding type B beta-lactamase in phage group II isolates of Staphylococcus aureus. *Antimicrob Agents Chemother*. 1998;42(12):3163-3168. doi:10.1128/AAC.42.12.3163

5. Song KH, Jung SI, Lee S, et al. Inoculum effect of methicillin-susceptible Staphylococcus aureus against broad-spectrum beta-lactam antibiotics. *Eur J Clin Microbiol Infect Dis Off Publ Eur Soc Clin Microbiol*. 2019;38(1):67-74. doi:10.1007/s10096-018-3392-6

6. Carvajal LP, Rincon S, Echeverri AM, et al. Novel Insights into the Classification of Staphylococcal β-Lactamases in Relation to the Cefazolin Inoculum Effect. *Antimicrob Agents Chemother*. 2020;64(5). doi:10.1128/AAC.02511-19

7. Ankrum A, Hall BG. Population Dynamics of Staphylococcus aureus in Cystic Fibrosis Patients To Determine Transmission Events by Use of Whole-Genome Sequencing. *J Clin Microbiol*. 2017;55(7):2143-2152. doi:10.1128/JCM.00164-17

8. Bernardy EE, Petit RA, Raghuram V, Alexander AM, Read TD, Goldberg JB. Genotypic and Phenotypic Diversity of Staphylococcus aureus Isolates from Cystic Fibrosis Patient Lung Infections and Their Interactions with Pseudomonas aeruginosa. *mBio*. 11(3):e00735-20. doi:10.1128/mBio.00735-20

9. Manara S, Pasolli E, Dolce D, et al. Whole-genome epidemiology, characterisation, and phylogenetic reconstruction of Staphylococcus aureus strains in a paediatric hospital. *Genome Med*. 2018;10(1):82. doi:10.1186/s13073-018-0593-7

10. Bolger AM, Lohse M, Usadel B. Trimmomatic: a flexible trimmer for Illumina sequence data. *Bioinforma Oxf Engl*. 2014;30(15):2114-2120. doi:10.1093/bioinformatics/btu170

11. Wick RR, Judd LM, Gorrie CL, Holt KE. Unicycler: Resolving bacterial genome assemblies from short and long sequencing reads. *PLoS Comput Biol*. 2017;13(6):e1005595. doi:10.1371/journal.pcbi.1005595

12. Souvorov A, Agarwala R, Lipman DJ. SKESA: strategic k-mer extension for scrupulous assemblies. *Genome Biol*. 2018;19(1):153. doi:10.1186/s13059-018-1540-z

13. Schwengers O, Jelonek L, Dieckmann MA, Beyvers S, Blom J, Goesmann A. Bakta: rapid and standardized annotation of bacterial genomes via alignment-free sequence identification. *Microb Genomics*. 2021;7(11):000685. doi:10.1099/mgen.0.000685

14. Feldgarden M, Brover V, Gonzalez-Escalona N, et al. AMRFinderPlus and the Reference Gene Catalog facilitate examination of the genomic links among antimicrobial resistance, stress response, and virulence. *Sci Rep*. 2021;11(1):12728. doi:10.1038/s41598-021-91456-0

15. Alcock BP, Raphenya AR, Lau TTY, et al. CARD 2020: antibiotic resistome surveillance with the comprehensive antibiotic resistance database. *Nucleic Acids Res*. 2020;48(D1):D517-D525. doi:10.1093/nar/gkz935

16. Bortolaia V, Kaas RS, Ruppe E, et al. ResFinder 4.0 for predictions of phenotypes from genotypes. *J Antimicrob Chemother*. 2020;75(12):3491-3500. doi:10.1093/jac/dkaa345

17. Katoh K, Standley DM. MAFFT multiple sequence alignment software version 7: improvements in performance and usability. *Mol Biol Evol*. 2013;30(4):772-780. doi:10.1093/molbev/mst010

18. Woo TE, Lim R, Surette MG, et al. Epidemiology and natural history of Pseudomonas aeruginosa airway infections in non-cystic fibrosis bronchiectasis. *ERJ Open Res*. 2018;4(2). doi:10.1183/23120541.00162-2017

19. Mulvey MR, Chui L, Ismail J, et al. Development of a Canadian standardized protocol for subtyping methicillin-resistant Staphylococcus aureus using pulsed-field gel electrophoresis. *J Clin Microbiol*. 2001;39(10):3481-3485. doi:10.1128/JCM.39.10.3481-3485.2001

20. Struelens MJ, Deplano A, Godard C, Maes N, Serruys E. Epidemiologic typing and delineation of genetic relatedness of methicillin-resistant Staphylococcus aureus by macrorestriction analysis of genomic DNA by using pulsed-field gel electrophoresis. *J Clin Microbiol*. 1992;30(10):2599-2605. doi:10.1128/jcm.30.10.2599-2605.1992

21. Gupta A, Jordan IK, Rishishwar L. stringMLST: a fast k-mer based tool for multilocus sequence typing. *Bioinforma Oxf Engl*. 2017;33(1):119-121. doi:10.1093/bioinformatics/btw586

22. Lam JC, Somayaji R, Surette MG, Rabin HR, Parkins MD. Reduction in Pseudomonas aeruginosa sputum density during a cystic fibrosis pulmonary exacerbation does not predict clinical response. *BMC Infect Dis*. 2015;15:145. doi:10.1186/s12879-015-0856-5
