# Supplementary figures and images for: Epidemiology and impact of methicillin-sensitive Staphylococcus aureus with β-lactam antibiotic inoculum effects in adults with cystic fibrosis
Source: Antimicrob Agents Chemother. 2023 Nov 15;67(12):e00136-23. doi: 10.1128/aac.00136-23 (PMC10720481; doi:10.1128/aac.00136-23)

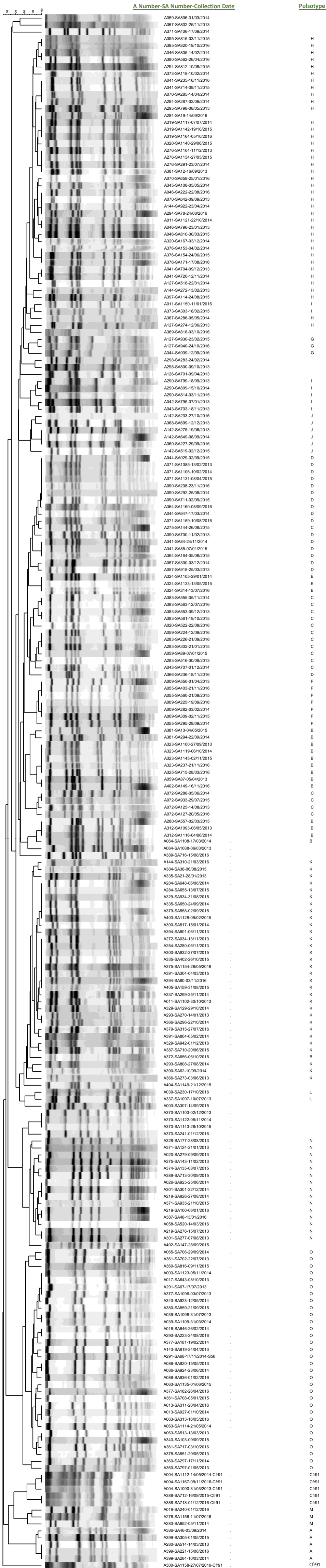

Supplement: Supplemental file 2 — Supplemental Figure E1. [file aac.00136-23-s0002.pdf]
